# Effects of Lifestyle Modification on Telomerase Gene Expression in Hypertensive Patients: A Pilot Trial of Stress Reduction and Health Education Programs in African Americans

**DOI:** 10.1371/journal.pone.0142689

**Published:** 2015-11-16

**Authors:** Shanthi Duraimani, Robert H. Schneider, Otelio S. Randall, Sanford I. Nidich, Shichen Xu, Muluemebet Ketete, Maxwell A. Rainforth, Carolyn Gaylord-King, John W. Salerno, John Fagan

**Affiliations:** 1 Center for Natural Medicine and Prevention, Maharishi University of Management Research Institute, Maharishi Vedic City, Iowa, United States of America; 2 Department of Physiology and Health, Maharishi University of Management, Fairfield, Iowa, United States of America; 3 MUM Molecular Biology Laboratory, Maharishi University of Management, Fairfield Iowa, United States of America; 4 Howard University College of Medicine, Department of Internal Medicine, Division of Cardiology, Washington DC, United States of America; Medical University of Graz, AUSTRIA

## Abstract

**Background:**

African Americans suffer from disproportionately high rates of hypertension and cardiovascular disease. Psychosocial stress, lifestyle and telomere dysfunction contribute to the pathogenesis of hypertension and cardiovascular disease. This study evaluated effects of stress reduction and lifestyle modification on blood pressure, telomerase gene expression and lifestyle factors in African Americans.

**Methods:**

Forty-eight African American men and women with stage I hypertension who participated in a larger randomized controlled trial volunteered for this substudy. These subjects participated in either stress reduction with the Transcendental Meditation technique and a basic health education course (SR) or an extensive health education program (EHE) for 16 weeks. Primary outcomes were telomerase gene expression (*hTERT* and *hTR*) and clinic blood pressure. Secondary outcomes included lifestyle-related factors. Data were analyzed for within-group and between-group changes.

**Results:**

Both groups showed increases in the two measures of telomerase gene expression, *hTR* mRNA levels (SR: *p*< 0.001; EHE: *p*< 0.001) and *hTERT* mRNA levels (SR: *p* = 0.055; EHE: *p*< 0.002). However, no statistically significant between-group changes were observed. Both groups showed reductions in systolic BP. Adjusted changes were SR = -5.7 mm Hg, *p*< 0.01; EHE = -9.0 mm Hg, *p* < 0.001 with no statistically significant difference between group difference. There was a significant reduction in diastolic BP in the EHE group (-5.3 mm Hg, *p*< 0.001) but not in SR (-1.2 mm Hg, *p* = 0.42); the between-group difference was significant (*p* = 0.04). The EHE group showed a greater number of changes in lifestyle behaviors.

**Conclusion:**

In this pilot trial, both stress reduction (Transcendental Meditation technique plus health education) and extensive health education groups demonstrated increased telomerase gene expression and reduced BP. The association between increased telomerase gene expression and reduced BP observed in this high-risk population suggest hypotheses that telomerase gene expression may either be a biomarker for reduced BP or a mechanism by which stress reduction and lifestyle modification reduces BP.

**Trial Registration:**

ClinicalTrials.gov NCT00681200

## Introduction

Hypertension is a major risk factor for cardiovascular disease (CVD) [1,2] There are racial/ethnic disparities in the prevalence, severity and clinical consequences of hypertension. The prevalence of hypertension is 48% higher in African Americans than white Americans [3]. This may contribute to the 50% higher mortality rate from cardiovascular disease in African Americans compared to whites [4,5]. There is evidence that psychosocial and environmental stress contribute to these disproportionate rates of hypertension and CVD in African Americans [6,7,8].

Amongst a range of proposed mechanisms, telomere dysfunction is emerging as a potential pathophysiological mechanism for hypertension and CVD [9,10,11,12]. For example, results from the Framingham Heart Study showed reduced leukocyte telomere length in individuals with a higher renin-to-aldosterone ratio, especially in patients with hypertension [10]. A five-year prospective study demonstrated that hypertensive patients who developed coronary artery disease (CAD) had shorter telomeres than matched patients who did not develop CAD. It proposed that telomere length is an independent risk factor for hypertension and CVD [11]. In an animal model, a direct link between telomerase activity and hypertension was reported. It was found that mice without telomerase displayed hypertension associated with increased in plasma endothelin ET-1 levels due to endothelin-converting enzyme ECE-1 overexpression [13].

In parallel, high psychosocial stress has been causally associated with low telomerase and shorter telomere length [14]. The mechanisms linking psychosocial stress with low telomerase levels and short telomere length may include activation of the sympathetic nervous system (SNS) and hypothalamic-pituitary-adrenal (HPA) axis, inflammation and oxidative stress [14,15]. Therefore, it may be hypothesized that psychosocial stress contributes to hypertension, at least in part, through effects on neurophysiologic mechanisms which that influence telomerase and telomere length [16]

Meta-analyses of randomized controlled trials of stress reduction with the Transcendental Meditation (TM) technique report reduced blood pressure in African American and white subjects [17,18,19]. Previous studies on the effects of stress reduction, meditation and lifestyle modifications on telomere function have been conducted in patients with prostate cancer and normal subjects [20]. However, to our knowledge, there has been no published study to compare effects of stress reduction and lifestyle modification for hypertension on telomere biology and blood pressure in hypertensive patients. Further, no previous study has investigated these effects in a racial/ethnic population with disparities in hypertension and CVD.

Therefore, the overall objective was to conduct a pilot study to compare effects of a stress reduction-based program to an extensive health education for hypertension on telomerase gene expression, blood pressure, lifestyle and behavioral risk factors in a sample of African American hypertensive men and women.

## Materials and Methods

### Design

**Fig 1:**
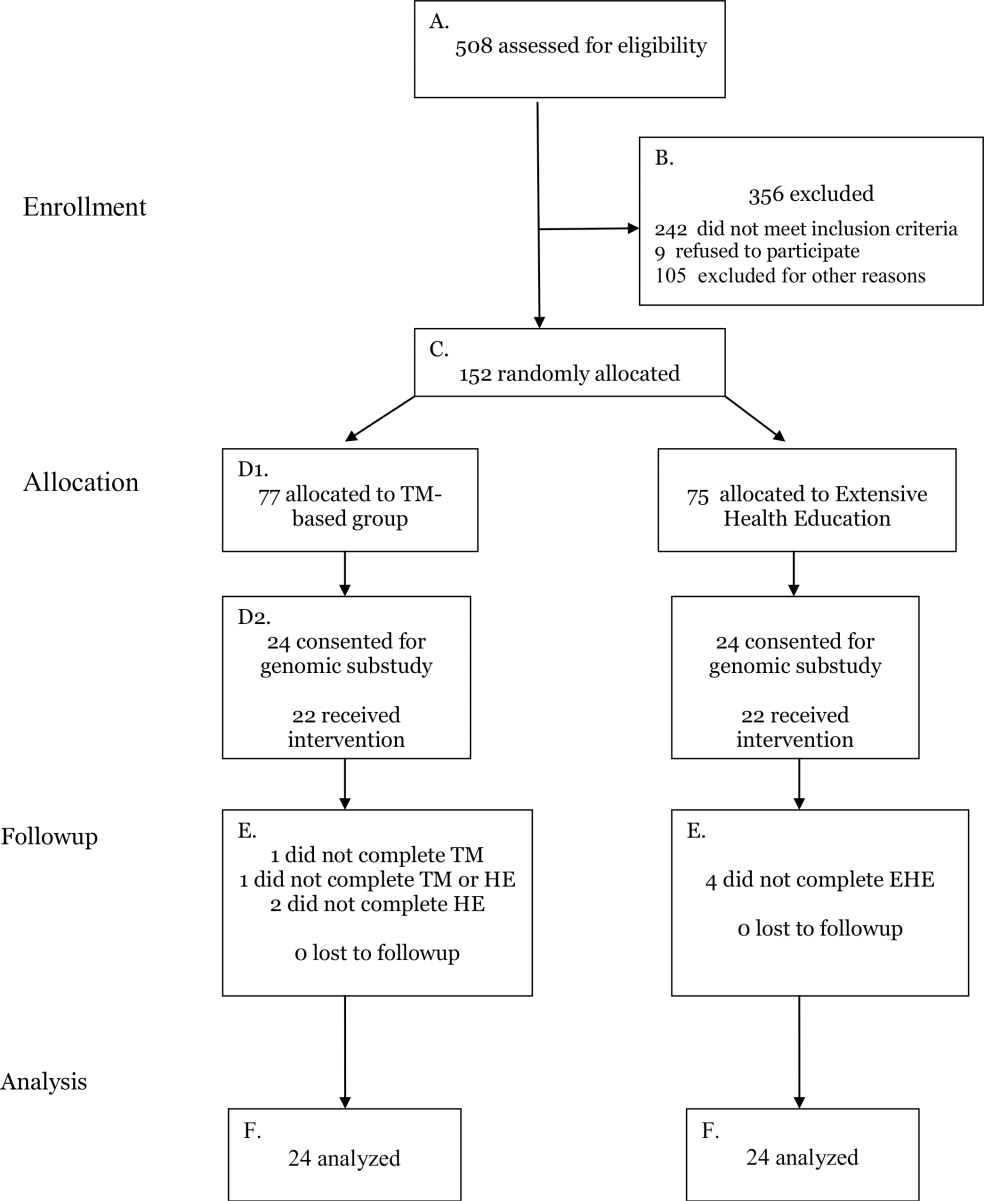
Participant Flow Diagram.

This study was part of a larger randomized controlled trial on physiological mechanisms of stress reduction and lifestyle modification for hypertension in African Americans. In the parent trial, 152 African American men and women with stage I hypertension were randomly assigned to a Transcendental Meditation-based program plus health education program (stress reduction, SR) or an extensive health education (EHE) program. As shown in the patient flow diagram (Fig 1), 48 of the 152 original randomized subjects (24 per group) volunteered for this ancillary study. All subjects were tested at baseline and after four months. Primary outcomes were expression of the telomerase genes: *hTR*, *hTERT* and clinic blood pressure. Secondary outcomes were psychosocial stress factors, dietary intake, body mass index, physical activity and telomere length.

The clinical site was Howard University Medical Center, Washington, DC. The study coordinating center was Maharishi University of Management Research Institute (MUMRI) in Maharishi Vedic City, Iowa. The institutional review boards of Howard University and Maharishi University of Management reviewed and approved the protocols in December 2007, December 2009, and April 2010. Subjects provided written informed consent twice. The first consent was for participation in the parent trial and the second consent was for participation in the telomere biology substudy study. Randomization of subjects into the study arms began in April 2008; data collection was completed in March 2012. There was a brief administrative delay for registration on ClinicalTrials.gov which occurred in May 2008. The overall trial was monitored by an independent data and safety monitoring board.

### Participants

Eligibility criteria included: age ≥ 40 years, male or female, stage I hypertension (SBP 140–159 mm Hg and /or DBP 90–99 mm Hg). Subjects who were taking two or more antihypertensive medications or sympatholytic medications i.e., beta blocker, alpha blocker, central adrenergic agent, ACE inhibitor were excluded. (The rationale for excluding subjects who required sympatholytics was that these classes of medications might have interfered with measures of sympathetic nervous system functioning used in the assessment battery for the parent study.) Subjects who had a history of major psychiatric disorders, dementia or substance abuse disorder, or any life threatening illness were excluded.

### Procedure

Subjects were recruited from Howard University Hospital clinics and through local media. Potential subjects who had a history of stage I hypertension but who were taking excluded antihypertensive medications were offered the opportunity to taper off these medications to determine if they were eligible according to BP criteria.

After baseline measurements in the parent trial, subjects were randomly allocated to the SR or EHE programs with stratification for antihypertensive medication status. There were three strata: no medication, diuretic only, or non-diuretic antihypertensive medication. Randomization was performed by the study biostatistician using computer generated random sequence. The study biostatistician concealed the allocation schedule and conveyed the assignments to the study coordinator. Investigators, data collectors and data management staff were blinded to group assignment. Intervention groups met separately to minimize contamination. This was a single blind protocol recommended for behavioral intervention trials [21]. Subjects were assessed at baseline and monthly for blood pressure (BP) and at baseline and four months for telomerase gene expression, body mass index (BMI), lifestyle behaviors (diet, physical activity and substance use), psychosocial distress factors, telomere length, and intervention adherence.

### Outcomes

#### Blood Pressure

Resting clinic blood pressure was measured in a standardized protocol by trained and certified observers using a Dinamap DPC 120X automated BP device (GE Medical Information Technologies, Chicago, IL). BP was measured three times at one-minute intervals in the seated position [22]. The first reading was discarded and the last two readings were averaged. BP was assessed at baseline and every month during the four-month follow up period.

#### Venipuncture

Venipuncture blood draws of 20 mL per subject at baseline and at posttest were taken by a trained phlebotomist at the Howard University General Research Center (GRC). Blood was collected in red labeled serum tubes without additives and frozen at -70° C on site. When all blood data was collected, frozen blood tubes were shipped on dry ice from Howard University in Washington, DC to the MUM Molecular Biology Laboratory in Fairfield, Iowa.

#### RNA Extraction

A total of 96 frozen blood clot samples (50–100 mg) were lysed in vials with 1mL of TRIZOL reagent (Ambion, AM9738). Homogenization was performed using a mortar and pestle, which was thoroughly washed first with RNAase ZAP solution (Ambion, AM9780), and then with DEPC treated water (Ambion, 4387937). The samples were then incubated at room temperature for 5–10 min. The homogenate was centrifuged at 12,000 g for 15 min at 4°C and the supernatant transferred to a new micro centrifuge tube. Next, 0.2 mL chloroform was added to the supernatant and after thorough vortexing, incubated at room temperature for 5–15 min. After centrifugation at 12,000 g for 15 min at 4°C, the upper aqueous phase was carefully removed, and transferred to new vials.

The RNA was precipitated with 0.5 mL isopropyl alcohol per 1mL of TRIZOL reagent and incubated at room temperature for 10 min. After centrifugation at 12,000 g for 8 min at 4°C the pellet was washed 2 times with 1 mL of 75% ethanol. The RNA pellet was dried at room temperature and re-suspended in 80 μL of the RNA storage solution (Ambion, AM7001).

In order to remove DNA, 50 μL of RNA sample was treated with 5μL 10x turbo reaction buffer and incubated at 37°C for 30 min. 5 μL of the DNase inactivation reagent were added and centrifuged at 10,000 x g for 3 min. 40 μL were removed and used for further analysis.

The purity of RNA was assessed by comparing light absorption at 260 nm versus 280 nm, and the integrity of RNA was verified by electrophoresis in 1% agarose gels, staining with ethidium bromide, and observation of well-defined 28S and 18S rRNA bands using the Gel Doc 1000/2000 image analysis system (BioRad, CA, USA).

#### Telomerase Gene Expression—hTR and hTERT mRNA Quantification by RT PCR

Telomerase is an RNA-dependent DNA polymerase that synthesizes telomeric DNA sequences. Activity of this enzyme is recognized almost universally as contributing to cellular proliferative potential [23]. Telomerase consists of two essential components: one is the functional RNA component (in humans called *hTR*), which serves as a template for telomeric DNA synthesis; the other is a catalytic protein (*hTERT*) with reverse transcriptase activity. Cells with elevated telomerase activity show delayed senescence and cellular aging [13,23].

Telomerase gene expression (*hTR*, *hTERT*) was determined by measurement of the levels of *hTR* and *hTERT* mRNA by the quantitative real time reverse transcriptase polymerase chain reaction (RT-PCR) (using the ABI Biosystem 7500 real-time PCR system. Analysis was carried out according to Yajima T *et al*.[24] with modifications of the *GAPDH* forward primer and the *hTERT* reverse primers (http://frodo.wi.mit.edu/primer3/) in order to obtain shorter PCR products.

Primers were synthesized by Integrated DNA Technologies, USA. PCR reactions (25μL) contained 12.5μL of 2x Quantifast RT PCR Mix (Qiagen multiplex Quantifast, 204954), 0.5 μL of 50x ROX dye solution, 1.25 μL of 20x primer-probe mix, 0.25μL of reverse transcriptase (RT) mix (QIAGEN OneStep RT-PCR Enzyme Mix), 0.5μL of RNase free water, 10 μL of template RNA or universal RNA (50 ng). The thermal cycling profile consisted of 20 min reverse transcription at 50°C, 5 min PCR initial activation step, at 95°C; 15 sec denaturation at 95°C, 15 sec annealing/extension at 60°C. Forty cycles of denaturation and annealing/extension were carried out.

To normalize for differences in the amount of total RNA added to each reaction, relative quantification of the *hTERT* and *hTR* mRNAs was carried out, using as internal reference amplification of the endogenous *GAPDH* mRNA. Nuclease free water was used as negative control, and universal RNA (Amsbio, R1234148-10) was used as positive control. Relative quantification measures the levels of the target gene mRNAs (*hTR and hTERT*) relative to the level of *GAPDH* mRNA.

#### Telomere Length Determination

An exploratory analysis of telomere length change was performed using the Bio-Rad MyiQ Single Color Real-Time PCR Detection System. Telomere primer pair telg and telc and scg albumin primers were synthesized by Integrated DNA technologies, USA (Table 1). The final concentrations of the telomere primer pair telg and telc, and scg albumin primer pair was 900 nM each in the master mix. PCR reactions were set up by aliquoting 12.5μL of QuantiFast SYBR Green master mix (Qiagen, 204057) and DNA sample (10μL), containing approximately 50ng of DNA diluted in pure water, for a final volume of 25μL per reaction. Three concentrations of reference DNA sample (Standard DNA) spanning an 81-fold range of DNA concentration were prepared in duplicate in every 96-well plate in this study. These reactions provided the data for the generation of the standard curves used for relative quantification. Inter and intra assay variability was adequate. DNA from all subjects, were run in duplicate.

**Table 1:** Primer and probe sequences.

*hTR* forward primer	FW: 5'-GGTGGTGGCCATTTTTTGTC-3'
*hTR* Reverse Primer	RV: 5'-CTAGAATGAACGGTGGAAGGC-3'
*hTR* Probe	probe 5'-FAM CGCGCTGTTTTTCTCGCTGACTTTC-3'
*hTERT* Forward Primer	FW: 5' -ACGGCGACATGGAGAACA A-3'
*hTERT* Reverse Primer	RV: 5'-GGGTCCTGAGGAAGGTTTTC-3'
*hTERT* Probe	probe: 5'-6-FAM-CTCCTGCGTTGGTGGATGATTTCTTGTTG-3'
*GAPDH* forward primer	FW: 5'-CAATGACCCCTTCATTGACC-3'
*GAPDH* reverse primer	RV: 5'-GAAGATGGTGATGGGATTTC-3'
*GAPDH* probe	probe 5'-Cy5-CAAGCTTCCCGTTCTCAGCC- 3'

*hTR*- Human Telomerase RNA Subunit; *hTERT*-human telomerase reverse transcriptase; GAPDH- glyceraldehydes 3-phosphate dehydrogenase

The thermal cycling profile consisted of Stage 1: 15 min at 95°C; Stage 2: 2 cycles of 15 sec at 94°C, 15 sec at 49°C; and Stage 3: 32 cycles of 15 sec at 94°C, 10 sec at 62°C, 15 sec at 74°C with signal acquisition, 10 sec at 84°C, 15 s at 88°C with signal acquisition. The 74°C reads provided the Ct values for the amplification of the telomere template (in early cycles when the scg signal is still at baseline); the 88°C reads provided the Ct values for the amplification of the scg template (at this temperature there is no signal from the telomere PCR product, because it is fully melted). Relative mean telomere length, or T/S ratio, was measured according to the protocol of Cawthon [25]. Relative T/S ratios reflect relative length differences in telomeric DNA only if the number of copies of S per cell that are effectively PCR-amplified is the same in all individuals being studied.

#### Behavioral and Lifestyle factors

BMI was calculated as weight/height^2^. Dietary patterns were assessed with the Block Dietary Food Consumption Questionnaire [26]. The Anger Expression (AX) scale was used to assess anger-in, anger-out, anger-control and total anger [27]. A modified Minnesota leisure time physical activity questionnaire was used for exercise [28].

### Interventions

The two interventions were matched for time, attention and other nonspecific factors. The stress reduction (SR) methods were the Transcendental Meditation (TM) technique. The TM technique was chosen as the stress reduction intervention for this study because of its standardization, reproducibility and previously demonstrated effects on physiological correlates of stress [29,30,31]. The TM technique is described as a simple, natural and effortless procedure that is practiced for 20 minutes twice a day while sitting with closed eyes [30,32,33]. During the practice, it has been reported that ordinary thinking processes settle down and a distinctive wakeful hypometabolic state characterized by neural coherence and physiological rest is achieved [29,33,34]. The teachers were African American and certified by Maharishi Foundation USA. Standard teaching materials and format were used. The instruction involved a standardized seven-step course comprising six 1.5–2 hour individual and group meetings over six days [30,32]. Follow up meetings were held twice a month for approximately one hour each for the duration of the 16 week intervention period (16 hours of stress reduction instruction and follow up). In addition, the stress reduction group received basic health education for hypertension recommended by the Seventh Joint National Commission on Detection, Evaluation and Treatment of High Blood Pressure (JNC VII) [35]. This supplementary class comprised eight sessions of one hour each, conducted every other week during the 16 week intervention period. The basic health education classes for the SR group were primarily didactic in format requiring no home practice, social support or other motivational reinforcements. Total intervention time for the SR group was 24 hours over 16 weeks.

The extensive health education (EHE) intervention actively taught JNC VII recommendations for lifestyle modification for hypertension [35]. The lifestyle modifications included weight reduction, dietary sodium restriction, and adoption of a Dietary Approaches to Stop Hypertension (DASH) type eating plan, regular physical activity and moderation in alcohol consumption [36]. The EHE group received additional substantial active reinforcement for lifestyle change through motivational videos, field trips and social support groups. The intervention comprised eight one-hour meetings and eight two-hour sessions during the course of the study. Subjects were encouraged to modify hypertension-related health behaviors in their daily lives. There was no stress reduction technique taught in the EHE intervention. Total intervention time for the EHE group was 24 hours over 16 weeks.

### Data Analysis

Baseline characteristics were compared with *t* tests for independent variables. Outcome data were assessed for within-group and between-group differences. Normality of distribution was tested by skewness and kurtosis. Normally distributed outcome data were tested for within-group differences by dependent *t* test and for between-group differences by analysis of covariance (ANCOVA) using baseline level as a covariate as well as variables on which there were significant baseline differences between the groups. Non-normally distributed data were tested for within-group differences by Wilcoxon matched pair test and for between-group differences by Kruskal Wallis ANOVA test. Statistical analyses were performed using SPSS software, Windows version 14.0 (IBM SPSS, Armonk, NY). All tests were two-tailed. Analyses were conducted according to the intention-to-treat principle.

## Results

The baseline characteristics of each group are shown in Table 2.

**Table 2:** Baseline Characteristics.

Parameters	SR groupMean ± SD	EHE groupMean ± SD
Age (years)	60.4±11.9	55.7±8.83
Gender (male)	45.8%	45.8%
Weight, kg	86.0 ± 15.5	89.4 ± 16.0
BMI, kg/m^2^	30.5 ± 4.5	30.7 ± 5.7
SBP, mm Hg	144.8 ± 4.2	146.7 ± 5.5
DBP, mm Hg	83.0 ± 1.4	87.5 ± 1.5
*hTERT*	1.23+2.76	0.81+1.25
*hTR*	1.50+1.81	4.21+6.00
Anger-in (-, 0–24)	5.7±4.2	6.1±4.0
Anger-out (-, 0–24)	6.9±3.0	6.1±1.9
Anger control (+,0–24)	14.2±3.5	13.1±2.8
Anger total (-,0–72)	22.3±7.4	24.3±7.7
Moderate activity (min/wk)	157.5±169.0	77.70±69.6
Vigorous activity (min/wk)	77.0±103.9	33.7±51.6
Carbohydrate (g)	233.1±136.9	174.9±67.8
Protein (g)	77.7±46.1	48.5±21.3
Fiber (g)	19.5±8.5	12.24±5.1
Sodium (mg)	2624.9±1459.5	1737.7±671.2
Total Calorie	1971.3±1383.8	1470.3±537.0
Total Fat (g)	93.2±66.4	60.6±23.0
Linoleic acid (g)	19.2±12.4	12.9±3.9
Oleic acid (g)	35.1±25.5	23.4±9.4
No Anti Hypertensive Medication	0.4±0.5	0.5 ±0.5
Diuretics only	0.2±0.4	0.3±0.5
ACE—Inhibitors	0.2±0.4	0.3±0.5
Angiotensin receptor antagonist	0.3±0.5	0.1±0.4
Beta blocker	0.04±0.2	0.2±0.4
Other antihypertensive medication	0.6±0.5	0.6±0.5

Abbreviations: BMI, body mass index (calculated as weight in kilograms divided by height in meters squared); SBP, systolic blood pressure; DBP, diastolic blood pressure; SR, stress reduction program; EHE, extensive health education group.

The groups were generally well matched; however, there were significant baseline differences in diastolic BP. When the baseline characteristics of the 48 subjects in this substudy were compared to the 104 subjects in the parent study, the results were generally similar except that the substudy group had a greater percentage of males (47.9% vs. 27.9%, p = .02) and a lower average BMI (30.0 vs. 32.4, p = .03) (data in S1 Table).

**Fig 2:**
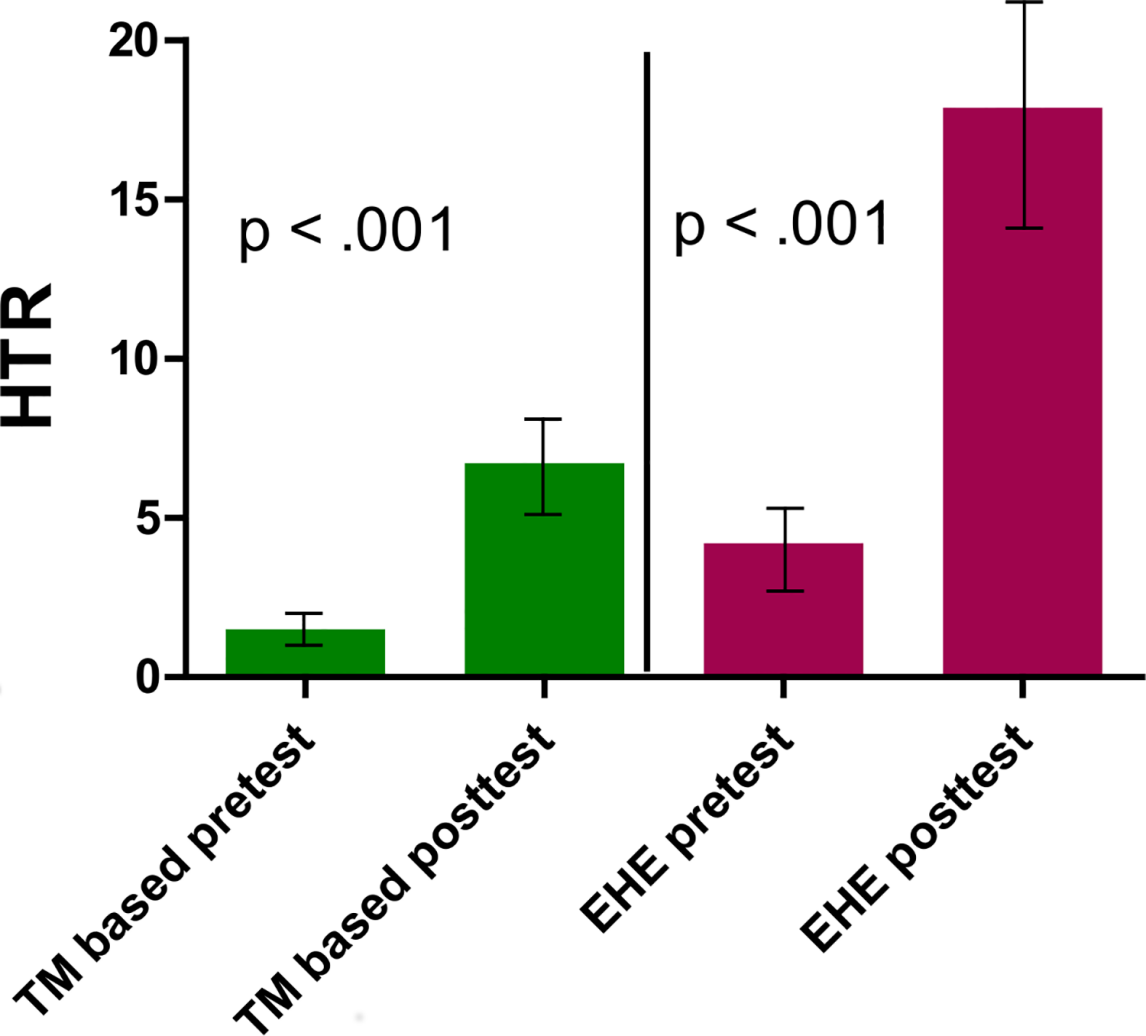
Pre-post changes in telomerase component (HTR) gene expression in the Stress Reduction (SR) and Extensive Health Education (EHE) groups.

**Fig 3:**
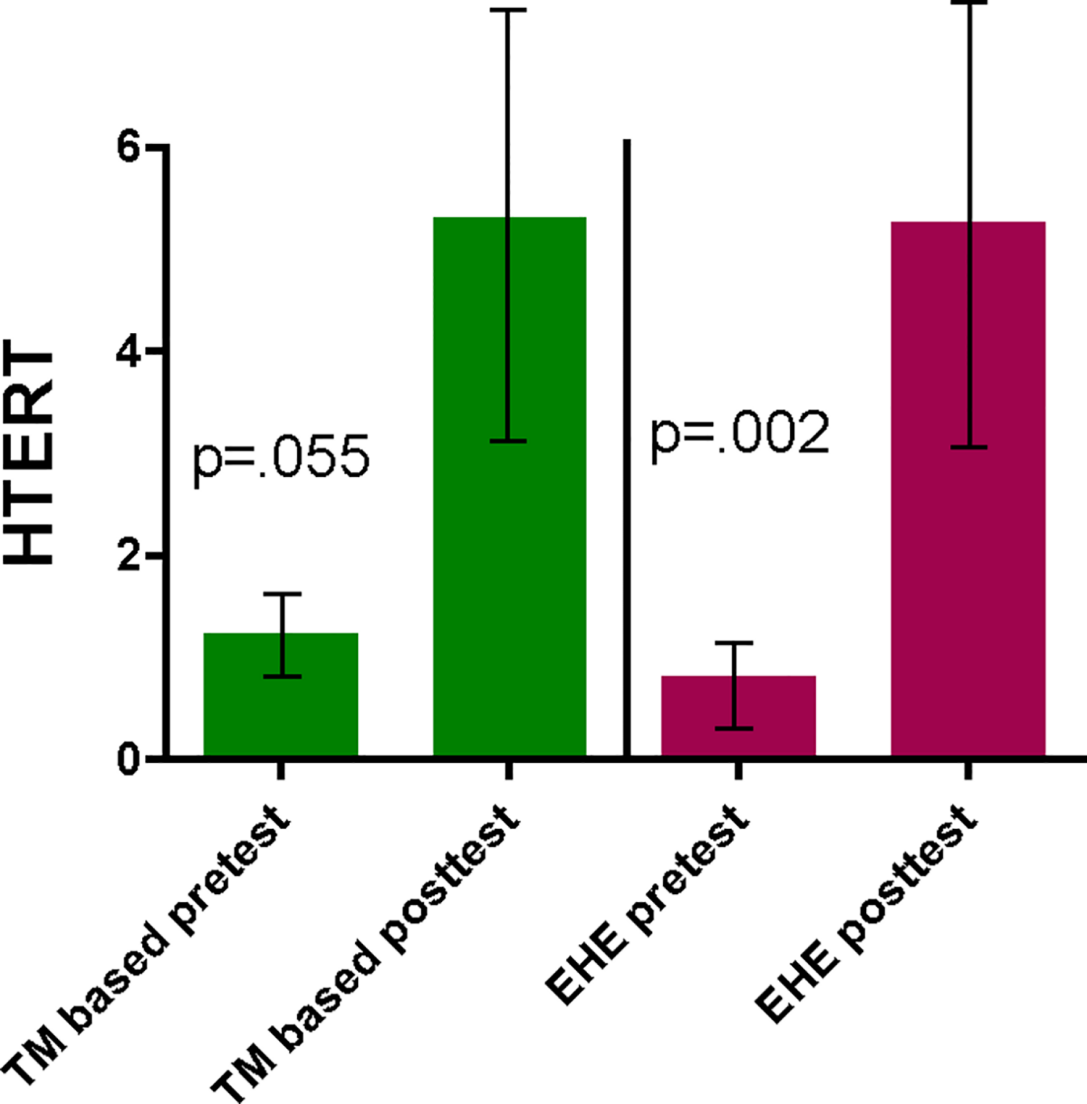
Pre-post changes in telomerase component (HTERT) gene expression in the Stress Reduction (SR) and Extensive Health Education (EHE) groups.

The telomerase gene expression results showed significant within-group changes in both the SR and EHE groups (Table 3) (Figs 2 and 3).

**Table 3:** Changes in Telomerase Gene Expression.

		SR group			EHE group	
	Median	Inter-quartile range	Within group significance p value	Median	Inter-quartile range	Within group significance p value
hTR	0.285	10.872	< 0.001	5.4	22.827	< 0.001
hTERT	0.035	1.576	0.055	0.600	4.325	0.002

Within group effects are calculated using Wilcoxon matched pair test; between group effects are calculated using Kruskal-Wallis ANOVA Test

The functional RNA component of telomerase, *hTR*, increased in both groups (*p* < .001 for SR and *p* < .001 for EHE). The catalytic component of telomerase, *hTERT*, increased in both the SR group (*p* = 0.055) and the EHE group (*p* = 0.002). There were no significant differences between the groups in *hTERT* or *hTR* gene expression.

As shown in Table 4, both groups showed significant within-group reductions in systolic BP. The adjusted changes were: SR = -5.7 mm Hg, *p* < 0.01; EHE = -9.0 mm Hg, *p*< 0.001).

**Table 4:** Changes in Blood Pressure.

Parameters	SR (mean ± SD)	Within group significance *p* value	EHE (mean ± SD)	Within group significance *p* value	Between group significance *p* value
SBP, mm Hg Unadjusted	-5.6 ±9.6	<0.01	-10.0 ±10.9	<0.001	
DBP, mm Hg Unadjusted	-0.9 ±5.4	0.42	-5.6 ±7.1	<0.001	
SBP, mm Hg Adjusted	-5.7 ± 10.8		-9.0 ±10.8		0.20
DBP, mm Hg Adjusted	-1.2 ± 6.4		-5.3 ±6.4		0.04

SD-Standard Deviation

There was no significant difference between groups in systolic BP reduction. For diastolic BP, the adjusted changes were: SR = -1.2 mm Hg, *p* = 0.42; EHE = -5.3 mm Hg, *p* <0.001). The between-group difference in diastolic BP change was significant (*p* = 0.04). A sensitivity analysis that included baseline age and physical activity in an ANCOVA yielded essentially the same results as the ANCOVA above with baseline BP as the covariate.

Table 5 shows changes in lifestyle-related factors.

**Table 5:** Secondary Outcome Changes.

Parameters	SR (Change mean score ±SD)	Within group significance*p* value	EHE (Change mean score ±SD)	Within group significance*p* value	Between group significance*p* value^†^
Weight, kg	-0.5 ±3.1	0.48	0.2 ±3.1	0.73	0.39
BMI, kg/m^2^	0.04 ±4.05	0.96	0.71 ±2.46	0.18	0.61
Anger-in (-, 0–24)	-1.0 ±3.3	0.17	-0.2 ±4.1	0.81	0.15
Anger-out (-, 0–24)	-1.0 ±2.5	0.09	-0.4 ±2.6	0.49	0.62
Anger control (+, 0–24)	-0.9 ±3.7	0.29	1.1 ±3.4	0.13	0.46
Anger total (-, 0–72)	-1.1 ±4.0	0.21	-1.7 ±7.6	0.29	0.64
Moderate activity (min/wk)	-31.7 ±118.3	0.27	-31.3 ±102.1	0.20	0.11
Vigorous activity (min/wk)	-25.0 ±86.9	0.24	-1.1 ±87.3	0.96	0.86
Sodium (mg)	-618.1 ±862.6	0.02	-343.4 ±421.0	0.02	0.62
Carbohydrate (g)	-50.4 ±106.0	0.10	-49.7 ±40.4	<0.01	0.64
Protein (g)	-13.9 ±20.8	0.03	-12.3 ±10.5	<0.01	0.70
Fiber (g)	-2.4 ±7.3	0.24	-1.3 ±4.0	0.31	0.88
Total Calorie (g)	-513.5 ±906.2	0.05	-432.9 ±276.9	<0.001	0.89
Oleic acid (g)	-9.4 ±21.0	0.12	-6.8 ±5.3	<0.01	0.73
Linoleic acid (g)	-5.1 ±9.8	0.07	-3.6 ±4.3	0.02	0.87
Total Fat (g)	-25.5 ±52.4	0.09	-17.0 ±14.3	<0.01	0.95

Direction of improvement: (+) a high score is desirable, (-) a low score is desirable^†^Based upon ANCOVA covarying for baseline measure and baseline diastolic blood pressure.

The SR group showed significant within-group reductions in dietary intake of sodium and protein. The EHE group reported significant within-group reductions in dietary intake of sodium, protein, carbohydrate, total calories, total fat, oleic acid and linoleic acid. There were no significant between-group differences in these lifestyle factors.

There were no significant within or between-group changes in telomere length (SR group = -0.05 ± 0.5 relative units; *p* value = 0.61 and EHE group = -0.02 ± 0.5 relative units; *p* value = 0.84; between group *p* value = 0.39).

Intervention meeting attendance was the first measure of compliance with the study interventions. In the stress reduction group, participants attended an average 87% of the Transcendental Meditation instructional meetings and 88.5% of the basic health education meetings over the course of 16 weeks. In the extensive health education group, participants attended 88% of the lifestyle modification education and support meetings over 16 weeks. Second, 92% of the SR group reported practicing the TM technique at home at least once per day. The average number of home practice sessions per day was 1.6.

## Discussion

Psychosocial stress, physical inactivity, obesity, excessive dietary sodium and alcohol intake may contribute to hypertension [37,38]. Psychological stress also induces inflammation and oxidative stress and activates the sympathetic nervous system and HPA axis [14,16,39]. Several previous trials of nonpharmacological interventions indicate that lifestyle modifications may reduce chronically elevated BP [37,40,41,42]. In the present study, we compared the efficacy of two behavioral lifestyle modification interventions for hypertension: a stress reduction-based program (SR) that included the Transcendental Meditation technique and second, an extensive health education program (EHE) in African American men and women with stage I hypertension. Both the stress reduction and extensive health education groups showed increased telomerase gene expression and reduced BP and over 16 weeks.

The reductions in systolic BP of 5–10 mm Hg in both groups are consistent with previously published literature reporting that conventional lifestyle modification (e.g. salt restriction, aerobic exercise, weight loss) and practice of the Transcendental Meditation technique are effective in lowering high BP with average changes in this range [17,18,19,40,41,42]. In this trial, systolic BP decreased in both the SR and EHE groups, although there was no significant difference in the change between groups. The EHE group showed a significant within-group reduction of -5 mm Hg in diastolic BP, while the reduction in the SR group was not significant (-1 mm Hg).

Both groups reported changes in lifestyle factors related to BP, including lower levels of self-reported sodium intake (300–600 mg/day) and protein (12–14 g/day); however the extensive health education group showed a greater number of dietary changes including reduced intakes of calories, carbohydrate, and fat than the stress reduction group. It may be that EHE group was motivated for more extensive dietary modifications due to active reinforcement of lifestyle change through the additional program of motivational videos, field trips and social support groups.

Many studies have found that African Americans patients are on average sensitive to the blood pressure-lowering effects of sodium intake [43]. Depending on the baseline blood pressure and degree of sodium intake of reduction, systolic blood pressure may be lowered by 4 to 8 mm Hg. A greater decrease in blood pressure is achieved when reduced sodium intake is combined with other lifestyle interventions [43,44]. In the SR group, practicing the TM technique may have reduced sympathetic nervous system activation which is associated with hypertension [45,46]. A number of studies demonstrate that the sympathetic nervous system may play a role in hypertension, in part by mediating salt sensitivity [45][47]. This is based on the findings that many salt-sensitive subjects have higher levels of norepinephrine and decreased dopamine. Norepinephrine is associated with sodium retention and dopamine promotes increased sodium excretion [45,47].

Concurrently, the SR and EHE groups each showed within-group increases in expression of the telomerase genes, *hTR and hTERT*. There were similar changes in both intervention groups. Telomerase RNA (*hTR*) along with telomerase reverse transcriptase protein (*hTERT*) is combined in vitro to reconstitute telomere synthesis. Hence, both these are necessary for telomere synthesis and are determinants of telomere length [48]. To support the importance of *hTERT* in telomere maintenance, tissue culture studies have demonstrated that introducing exogenous *hTERT* into primary cells results in the induction of telomere maintenance and escape of cells from senescence [49,50]. Introducing exogenous *hTERT* has also been shown to protect cells from stress-induced apoptosis and necrosis, thereby delaying cellular senescence and cell death [51]. The *hTR* component is also considered an essential requirement for telomere maintenance and telomerase activity in normal tissues and disease [23].

Clinical associations have been observed between low telomerase levels, short telomere length and hypertension. It has been hypothesized that telomere biology may play a pathophysiological role in the development of hypertension [9,10,11,12,13,52,53]. In this study, the two intervention groups exhibited both increased telomerase gene expression and reduced BP. These results are consistent with the hypotheses that either an increase in telomerase gene expression is a biomarker for effects of meditation and lifestyle modification in hypertension or that increases in telomerase gene expression may be part of the molecular pathway that causally lowers BP. Verification of whether this association of telomerase and BP is a biomarker or causal link requires considerably more experimental investigation.

Several previous studies have examined the relationship between telomerase, telomere length, BP and cardiovascular hemodynamics—although these did not include antihypertensive interventions[53]. Findings from Jeanclos *et al*.[54] showed that pulse pressure was inversely correlated with telomere length in white blood cells (WBCs), suggesting an inverse correlation between telomere length and pulse pressure. Furthermore, in a cross-sectional study comparing hypertensive and normotensives, it was shown that the systolic and diastolic blood pressure negatively correlated with relative telomere length [55]. In another study using a mouse model, it was shown that inducing changes in the phenotypic expression of vascular walls both predisposed to development of hypertension and resulted in shortening of telomere length [13]. A study by Benetos *et al* [56] found in multivariate analysis that telomere shortening was significantly associated with increased pulse pressure and pulse wave velocity in men.

Many studies have found that chronic psychosocial stress is associated with decreased telomerase activity [52,57,58]. Lifestyle modifications have been linked to increased telomerase activity. Previous research has reported effects of mind-body interventions on telomerase activity in nonhypertensive populations and/or did not report BP. Lavretsky *et al*. conducted a pilot trial with 39 dementia caregivers using meditation or a relaxation music control for eight weeks. Their results suggested increased telomerase activity in peripheral blood mononuclear cells (PBMC) in the experimental group compared to controls [59]. In another study, 30 participants in a three-month meditation retreat and showed higher levels of telomerase activity in their PBMCs compared to the wait-list controls [60]. Dusek *et al*. studied the effects of relaxation on genome-wide gene expression in a general population sample, but did not report *hTR* or *hTERT* RNA levels [61]. In addition to not examining or reporting relationships of gene expression, telomerase and cardiovascular outcomes (ie, BP), none of these studies focused on high risk racial/ethnic populations.

In practical clinical settings, concerns have been raised about compliance with lifestyle modification interventions [62,63,64]. For example, Hamer comments that, “Despite the wealth of evidence regarding the benefits of healthy lifestyle, the available data suggest that adherence to these recommendations among hypertensive patients is poor.” (Hamer, 2010)[62]. By contrast, 92% of the patients in the stress reduction group in this study reported practicing the Transcendental Meditation technique at least once per day at home. The average home practice frequency was 1.6 times per day. The adherence findings from this 16 week trial compare favorably with long-term adherence to TM practice reported over an average of five years in a trial of patients with coronary heart disease [65]. These data suggest that this form of stress reduction is clinically feasible with relatively high adherence. There was no significant change in physical activity in either group. Self reported changes in diet are discussed above.

There are several limitations of the present study. The first was the lack of an inactive control group. To address this limitation, future research might use a three-arm design, including an inactive or placebo control group. On the other hand, the current pilot trial may be considered a comparative effectiveness study of two clinical programs, both of which have been recommended for consideration in the treatment of hypertension [36,41,42,46,66]. The second limitation involved the use of multimodality therapies in each comparison group. This design however, reflects clinical practice pragmatics and enhances external validity of the study [67].

Another limitation was the relatively small sample size of 48. Although there were significant increases in *hTERT* and *hTR* gene expression within each of the two lifestyle modification groups, the results indicated that there were no significant differences between the two groups studied. A larger sample size would generate the statistical power needed to either confirm or disprove these findings.

There were no statistically significant differences in telomere length within or between groups. Telomere length is usually not sensitive to short-term interventions, such as the 16 weeks in the present study, but may require a year or more for detectable changes to be observed [68]. Thus, a longer term, prospective trial is needed to determine effects of antihypertensive lifestyle modifications, including stress reduction on telomere length.

We also did not find significant changes for anger expression either between or within groups. Recent meta-analysis of psychological effects of the Transcendental Meditation technique have reported reductions in anxiety [69,70] and a long term trial in CHD patients found reduced anger expression over an average of five years follow up [65]. It may be that anger is a less sensitive marker of meditation over the short-term compared to long-term follow up. Anxiety may be more sensitive short-term psychological marker, although it was not measured in the current study.

In this study, we have used whole blood samples to determine the telomerase activity. One of the risks of using whole blood samples is that the proportions of different types of white blood cells, in particular T cells and B cells, among subjects. It is known that these cell types vary in telomere length. Therefore, if the cell-type composition varies from subject to subject, it could affect the levels of measured telomerase [71] Various levels of hTR and hTERT have been observed to be upregulated when T and B cells are activated but to date this has been observed only in studies in which activation has been done for experimental purposes.[72]

The larger parent trial was a randomized controlled trial. However, this ancillary study comprised volunteers from each group after they had been randomized. Therefore, this pilot trial was not a randomized trial, although its sample was drawn from an RCT. The experimental design may be considered a quasi-randomized trial [73]. Future studies should prospectively employ a random allocation design to the study of stress reduction, lifestyle modification and telomere biology. Finally, this pilot trial was limited to a single racial/ethnic sample of African Americans. Follow up trials involving other populations would provide evidence about the generalizability of these findings.

In conclusion, this pilot trial compared a stress reduction program based on the Transcendental Meditation technique to an extensive health education intervention in African American patients with stage I hypertension. Both interventions demonstrated increased telomerase gene expression and reduced BP. The results suggest molecular biomarkers or mechanisms of these two lifestyle modification approaches in the treatment of hypertension.

## Supporting Information

S1 CONSORT Checklist CONSORT checklist of information to include when reporting a randomised trial. (DOC)

S1 Protocol Research Design and Methods for parent study. (DOCX)

S1 Table Inclusion of subjects who participated in the telomerase substudy compared to those who did not. (DOCX)
