# Supplementary material for: Effects of Lifestyle Modification on Telomerase Gene Expression in Hypertensive Patients: A Pilot Trial of Stress Reduction and Health Education Programs in African Americans
Source: PLoS One. 2015 Nov 16;10(11):e0142689. doi: 10.1371/journal.pone.0142689 (PMC4646647; doi:10.1371/journal.pone.0142689)
Supplement: S1 Protocol — (DOCX) [file pone.0142689.s002.docx]

**S1 Protocol**

# Research Design and Methods

## Overall Design

This study of physiological mechanisms of meditation in the treatment of hypertension in African Americans will be a Phase II mechanistic trial of 152 African American men and women aged 40 years and older with stage I hypertension randomly assigned to either the Transcendental Meditation program plus health education or a health education ‘alone’ control program for a study period of four months. The application of nonpharmacologic treatment in the management of stage I hypertension is consistent with national practice guidelines (5). All subjects for the study will have documented Stage I hypertension, i.e. SBP 140-159 mm Hg and/or DBP 90-99 mm Hg. Subjects will be tested at Howard University Clinical Research Center (CRC) for a total of three days of physiological and behavioral testing. As illustrated in the diagram below, on the first day of baseline testing, subjects will start ambulatory heart rate monitoring and ambulatory blood pressure monitoring for 24 hours. On the second day of testing, subjects will undergo psychological stress testing with mental arithmetic and cold pressor to assess hemodynamic, neurovascular, and neuroendocrine reactivity and recovery. On the third day of testing, subjects will undergo exercise stress testing. Dr. Randall, Co-PI, cardiologist, and clinical hypertension specialist, will monitor the BP medical status of subjects in the study. In addition, all subjects will continue their standard medical care with their usual health care providers.

## Timetables

### Overall Study Timetable

**0 – 6 months:** Startup Phase- IRB and subcontract approvals, staff hiring and training, equipment/supply purchasing, test form and operations manual development and protocol piloting.

**7 – 39 months:** Recruitment of subjects; testing, data entry and management

**40 – 42 months:** Posttesting, data entry and management

**43-48 months:** Data analysis and write up

### Testing Protocols: For Baseline and 4-Month Posttest

Subjects will be instructed to abstain from consuming food, caffeine, and smoking for two hours before testing

## Testing protocol – Day 1

|  | **24 hour collection** | **Administered in lab**  **(45-60 min period)** |
| --- | --- | --- |
| **Heart rate variability** | X |  |
| **Ambulatory blood pressure** | X |  |
| **Psychological and behavioral testing** |  | X |

## Testing protocol – Day 2

**Testing Protocol – Day 3**

## Subjects

All subjects will meet the following inclusion and exclusion criteria:

### Inclusion Criteria

1. Ethnicity: Self-identified as African American

2. Gender: Male or Female

3.Age: 40 years or older

4. Residence: Washington, DC or surrounding communities

5. Blood pressure: Stage I hypertension by JNC VII criteria (5) defined as systolic BP of 140-159 mm Hg and/or diastolic BP of 90-99 mm Hg average without antihypertensive medication documented in the study clinic on two or more occasions; or BP within this range with diuretic only. Note: the rationale for excluding subjects who require other antihypertensive medications (e.g. beta blockers, ACE inhibitors, alpha blockers, central adrenergic agents) is that these classes of medications may interfere with the sensitive measures of SNS functioning used in the outcomes assessment.

### Exclusion Criteria

1. Blood pressure: <140/90 mm Hg or ≥ 160/100 mm Hg
2. A history of clinical cardiovascular disease (defined as myocardial infarction, angina, intermittent claudication, congestive heart failure, and stroke) or chronic renal failure
3. Two or more antihypertensive medications or sympatholytic antihypertensive monotherapy, e.g. beta blocker, alpha blocker, central adrenergic agent, ACE inhibitor
4. Any other life threatening illness, e.g. advanced malignancy
5. History of major psychiatric disorder, e.g. psychosis, dementia or substance abuse disorder
6. Inability or unwillingness to give informed consent

### Subject Availability and Recruitment

Subjects who meet the above eligibility requirements will be recruited from specialized clinics at Howard University Medical Center in Washington, DC, including Dr. Randall's hypertension clinic, medicine, family practice, and private physicians’ clinics. In addition, prospective subjects will be recruited every month throughout the study from primary care physician referrals, churches, health fairs, community-wide high blood pressure screening events, radio announcements, and newspaper advertisements. As noted above, all subjects will have stage I hypertension documented during a series of three baseline visits. Subjects who are interested in participating in the proposed study and who have a history of stage I hypertension but who are taking antihypertensive medications will be offered the opportunity to taper off these medications to determine if they are eligible by BP criteria for the study. Co-PI Dr. Randall, cardiologist and hypertension specialist, will medically supervise any antihypertensive medication changes in study subjects. In addition, Dr. Randall will closely monitor the BP status of all subjects during the course of the study. (cf. Section E.1.1.1– Human Subjects Involvement and Characteristics for detailed BP management protocol).

The recruitment procedures for the current study have been employed successfully to meet or exceed enrollment targets in previous NIH-supported clinical trials of CVD prevention with behavioral interventions conducted by Drs. Schneider, Nidich, Randall, and colleagues. In order to fulfill the recruitment target of 152 enrolled subjects (an average of 4.6 subjects/month), we plan an intensive program to screen approximately 7600 African American men and women over a 33 month period (or about 230 individuals/month). Of these, we estimate, based on previous clinical trial recruitment experience, that about 14 individuals per month will meet the study eligibility criteria. Of these 14 per month, it is estimated that an average of 4.6 per month will complete the informed consent and three days of baseline testing required for randomization into the treatment arms of the study. Based upon the large availability of potential subjects in the greater Washington, DC area and major interest within the target population in nonpharmacologic methods for hypertension and stress reduction in particular, it is anticipated that recruitment for the proposed study will be successfully accomplished within the revised timeframe.

### Randomization

Simple randomization (without stratification) will take place after determination of eligibility and completion of baseline testing and will be performed by the study biostatistician.

### Blinding

All data collection, data management, and data entry staff will be blinded to treatment assignment of subjects. In addition, primary care physicians will be blinded to the treatment allocation of the subjects. These physicians will be informed that the subjects are participating in a study of lifestyle modification with two different interventions but will not be informed of the specific allocation of the individual subjects. Also, participants will be instructed not to declare their treatment grouping to their usual health care providers. However, if it is necessary for medical treatment of the participant, which is anticipated to be an unusual situation, physicians may ask their patients about which adjunctive lifestyle modification they are participating in, or contact the project coordinator for further information. Since the data collection will be blinded, yet participants will necessarily be aware of their treatment status, this will be a single blind study (150). None of the data collection staff will be involved in data analysis. As described below, endpoint data collected at the field site in Washington, DC will be coded with subject ID# only, copied, and sent to the Data Management and Biostatistical unit at the Institute for Natural Medicine and Prevention for data processing. All data will be entered independently into the unit’s MS ACCESS database in preparation for final statistical analysis.

## Outcomes Assessment

### Cardiovascular Hemodynamics

#### Clinic Blood Pressure

Resting clinic blood pressure (casual BP) will be measured in a standardized fashion by trained and certified observers using a mercury sphygmomanometer (5). After resting 5 minutes the BP will be evaluated with a proper sized cuff in the right arm at the heart level 3 times. The average BP of the 2^nd^ and 3^rd^ reading will be counted. The process will be repeated 3 times at 5-7 days intervals. The average from the 2^nd^ and 3^rd^ visits will be used as baseline BP for eligibility purposes. Also the average of the 2^nd^ and 3^rd^ readings at **4**-month posttest will be used to determine the primary BP outcome. BP will be assessed at baseline, I, 2, **3 and 4** months posttesting.

#### Cardiac Output, Stroke Volume, Total Peripheral Resistance

Hemodynamic data will be collected using standardized published protocols (52, 65). Systolic BP and diastolic BP will be monitored with a Finometer monitor described below. Cardiac output (CO), heart rate (HR), and stroke volume (SV) measurements will be obtained using a GE VIVID 5 ultrasound machine. Doppler-derived CO measurements have been shown to provide absolute measurements of CO and stroke volume (52, 65). Doppler examination will be conducted using a continuous-wave transducer placed in the suprasternal notch and directed toward the aortic valve. The beam is directed parallel to aortic flow to measure flow velocity. Utilizing computer analysis and the area under the velocity curve (flow velocity integral), stroke volume and CO are calculated. Blood pressure and CO values measured simultaneously are used to calculate total peripheral resistance (TPR={SBP + 2*DBP}/3/CO). Pulse pressure will be determined from cuff SBP –DBP. Cardiac output, stroke volume, and total peripheral resistance (TPR) will be assessed at baseline and **4** months posttest.

#### Arterial Compliance

Arterial compliance (ΔV/ΔP) is an important component of input impedance that describes the characteristics of the arterial system and determines the left ventricular afterload. The compliance and resistance components of input impedance are the major hemodynamic determinants of BP and both can be altered by sympathetic nervous system tone and structural vascular changes. The ratio of stroke volume to pulse pressure (SV/PP) was shown to be a reliable method to determine arterial compliance and to detect changes in it (148). SV will be measured and calculated from ultrasound images as described above. Arterial compliance will be assessed at baseline and **4** months posttest.

#### Left Ventricular Mass

#### We will assess LV mass because it is an independent predictor of CVD morbidity and mortality. Echocardiography will be performed according to a standard protocol by a qualified sonographer at Howard University Hospital. All subjects will undergo echocardiography at baseline and 4 months posttest.

#### Echocardiograms will be obtained with participants lying in a modified left lateral decubitus position with head angled at 30 degrees from the horizontal., Recordings will be made at end expiration using a sonographic recorder with a 2.5/3.5 MHz transducer. Strip chart recordings of echocardiographic data will be made on a fiber optic L585 Honeywell recorder or similar model on light sensitive paper at 50 mm/s. Two-dimensional (2D) studies will be recorded using 1/2 inch VHS videotape. A 2D parasternal long axis view of the left ventricle will be obtained to adjust M-mode cursor position perpendicular to the interventricular septum and posterior wall of the left ventricle at the mitral valve chordal level. Following this, the transducer will be rotated 90 degrees until a satisfactory 2D short axis view of the LV wall at the chordal level is obtained, ensuring that the M-mode will be centered and perpendicular to the LV septal and posterior walls.

#### LV measurements will be obtained at end-diastole. End diastolic measurement criteria will be based on the Penn convention. The Penn convention excludes endocardial for epicardial surfaces in the measurement of wall thickness and included endocardial surfaces in the LV dimension measurement (Devereux & Reichek, 1977). End-diastole is defined as the peak of the R-wave of the QRS complex. The other standard in the field is the American Society for Echocardiography (ASE) convention. The results from these two methods are very highly correlated. Due to the constraints of the echocardiography facilities, there will only be time to use one convention. It has suggested use of the Penn convention, which provides a more reliable estimate of left ventricular mass (Devereux & Reichek, 1977).

#### The LV measurements will include interventricular septal thickness at end diastole (IVSTd), the posterior wall thickness at end-diastole (PWTd), and left ventricular internal dimension at end-diastole (LVID). From the diastolic measurements, LV wall mass (LVM) will be calculated from the Penn convention, according to the equation of Devereux and Reichek (1977) by the formula LVM = 1.04(IVST + PWT + LVID)^3^) - 13.6 gm. LV mass index (LVMI g/m^2^) will be calculated by dividing LV mass by body surface area (BSA). BSA (m^2^) = 71.84 (Height (cm))0.725 + (Weight (kg))0.425. A second index (LVMI g/m) will be calculated dividing LV mass by height (Levy et al., l987). LV cross section area LVCSA (cm^2^) = *pi* (0.5(IVSTd + PWTd + LVIDd)2 _ 0.5 (LVIDd)^2^) (Ditchey et al., 1981) will also be calculated using ASE criteria. Relative wall thickness will be calculated as 2PWTd/LVIDd. LV diastolic dimensions index will be calculated as LVIDd/BSA.

#### Diagnostic criteria for LV hypertrophy using LV mass index (g/m2) will be ≥ 110 g/m2 for men and ≥ 100 g/m2 men for women, representing the approximate sex-specific 60th percentiles of a previously published reference standard in a normal population (Devereux et al., 1984) both based on Penn convention measurements.

##### Echocardiography reading and quality control: For the echocardiography studies, the observer rater will be blinded to the treatment status of the subjects.

####

### Catecholamines

Blood samples will be obtained during the baseline rest, mental arithmetic, and cold pressor stress testing for plasma levels of norepinephrine and epinephrine. A minimum of 20 minutes of rest will follow insertion of the cannula prior to beginning baselines sampling. Blood samples will be withdrawn during the final minute of baseline rest and each mental stress task. Blood will be sampled through a flexible cannula inserted into a forearm vein, attached to polyethylene tubing, with potency maintained by continuous, slow rate saline infusion (ND, 0.9% NaCl). Blood samples for catecholamines will be collected in EDTA-coated tubes, immediately cold centrifuged, pipetted for plasma extraction, stored in triplicate and frozen at –70 degrees Centigrade. Plasma norepinephrine (NE) and epinephrine (Epi) will be assayed using a standard high performance liquid chromatography assay through the Howard General Clinical Research Center (GCRC) laboratory services utilizing(Quest Diagnostics). Plasma catecholamines will be assessed at baseline and **4**-month posttesting.

In addition to these laboratory measures, 12 hour overnight urine collections for NE and Epi will also be assayed for each subject at baseline and 4 month posttesting.

### Cardiovascular Reactivity Testing

#### Measurement of Blood Pressure and Heart Rate in the Laboratory

Blood pressure and heart rate will be collected during the laboratory stressor periods using a TNO Finometer Continuous Blood Pressure Monitor, which takes beat-to-beat pressures in a non-invasive manner, using the Peñaz method (151) and which allows the BPs to be read and stored electronically. SBP and DBP, as well as HR, will be measured. This method uses a finger cuff, worn on the third finger of the non-dominant hand. The method of operation of its predecessor, the Finapres, has been described in detail (152-155). The TNO Finometer is manufactured by the research organization (BMI/TNO, Netherlands) that developed the Finapres 2300. The Finometer employs the identical measuring algorithms as the Finapres; thus, the reliability and validity data collected on the Finapres apply to the Finometer as well (156). It has been demonstrated as a useful alternative to intra-arterial BP measurement in laboratory testing (157), as well as in clinical practice (158). In addition, it has been shown to track intra-arterial readings extremely well, even during sudden BP changes (157), making it a good candidate for use during laboratory testing. Finally, members of the present collaborative team have reported that the BP and HR measurements are extremely reliable, due to the large number of measurements that can be collected (159). The TNO Finometer/Finapres has been validated, and has met the criteria set out by the American Association of Medical Instrumentation (155). Data are downloaded via cable into a PC and stored using dedicated software. This method will be used to measure BP and heart rate during the two days of reactivity protocols described in detail below. These measures will be assessed at baseline and **4**-month posttesting.

#### Reactivity Testing

Following instrumentation for blood pressure and heart rate as described above, the subjects will be placed in a supine position. Following baseline evaluation, during both the baseline and posttest evaluations, the stressors will be presented as described in detail in Section D.4.2.4. A 30-minute reclining supine rest period will precede the administration of the first stressor. For the mental arithmetic challenge test, blood pressure and heart rate readings will be obtained at 1, 3 and 5 minutes and then every other minute during the 10-minute recovery period. These same measures will be obtained during the cold pressor task at 30 minutes and 90 minutes and then every 2 minutes during the 10-minute recovery period. For the treadmill exercise task, these same measures will be obtained every 3 minutes and then every 2 minutes during the 10-minute recovery period. Hemodynamic data are reduced by computing mean scores for measures obtained during the initial supine rest and all prestressor periods. Reactivity change scores are then calculated by subtracting mean values obtained during the prestressor period immediately preceding the stressor from the peak reading obtained during the stressor. Recovery from each laboratory stressor will be assessed over a 10-minute period.

#### Measurement of Cardiovascular Recovery

BP recovery is inherently a dynamic process, not a stable state. The current collaborative team has examined several approaches to assessing recovery, including 1) amount of time for BP to return to pre-stress provocation resting levels. A problem with this method is that many subjects do not ever return to baseline levels within the session. 2) A change score in which BP level at some fixed point during recovery is subtracted from the pre-stress provocation baseline. A problem with this method, however, is that the selection of the recovery time point is arbitrary. 3) The arithmetic difference (change score) between the mean BP level during the recovery period and during the baseline period. While intuitively appealing, this measure may be heavily influenced by baseline and reactivity levels, and may oversimplify the dynamic recovery processes. Other methods of measuring recovery have been used, but all have similar shortcomings; we have reviewed these extensively in a recent paper (92).

Dr. Gerin of the collaborative team has recently published an approach based on fitting a 3-parameter mathematical curve to the series of BP readings taken during recovery (103). This technique produced high test-retest reliability in a population of normotensive college students, and provides a means of tapping the dynamic nature of post-stress recovery. In addition, this method has been shown (103) to have reliability superior to the three methods noted above. Thus, in the proposed study we will assess BP recovery using this curve-fitting technique (see Data Analysis section for further details).

#### Selection of Psychological and Physical Stressor Battery

A battery of stressor variables has been recommended to assess cardiovascular reactivity (43, 46, 86) in order to assess potentially different physiological mechanisms of hypertension. Conceptually, three distinct classes of stressor variables may be delineated: 1) psychological stressors; 2) passive psycho-physical stressors; and 3) active physical stressors (43). These tasks not only differ in cognitive components but also may stimulate differing elements within the autonomic nervous system (cf. Background and Significance) (43, 46). This mechanistic trial will employ 3 widely-used and standardized cardiovascular reactivity stressors, involving psychological stress tasks (mental arithmetic and the passive psycho-physical stressor (cold pressor test) (160, 161) and an active physical stress task (aerobic exercise (43). During the second day of testing, mental arithmetic and cold pressor test stressors will be administered. The third day of testing will employ treadmill exercise. The protocol will be the same for each laboratory stressor component: 1) a baseline period, 2) instruction/anticipation of stressor, 3) reactivity to onset of stressor, and 4) recovery period. This same sequence for all 3 stressors will be repeated at **4**-month posttesting.

#### Stressor Task 1: Mental Arithmetic

Following baseline evaluations, the psychological challenge stressor (mental arithmetic) will be presented to each subject during a 2-minute instructional phase. Subjects will be instructed to count aloud backward by 13, starting at the number 1079, as quickly as possible. Throughout the 5-minute task subjects will be told by the experimenter to speed up and be accurate (76, 102). This task has been found to have a pronounced effect on blood pressure and heart rate and subject self-ratings indicate that it is frustrating and anger-arousing (102). After the administration of the stressor, there will be a 10 minute recovery period.

#### Stressor Task 2: Cold Pressor Test

After a 10-minute recovery period to the mental arithmetic stressor and an additional 5-minute baseline period, subjects will be instructed in the cold pressor test and informed of the often times painful and difficult nature of the experience (90 seconds). Following this instruction, a plastic bag containing 6 cups of crushed ice and 1.5 cups of water (bag temperature 3-4°C) will be applied to the subject’s forehead for approximately 2 minutes. A 10 minute recovery period will follow (162).

#### Stressor Task 3: Treadmill Exercise

The third laboratory stressor will be aerobic exercise (treadmill testing), administered on a separate day from the first two stressors (mental arithmetic and cold pressor test). Patients will be instructed not to eat or drink caffeinated beverages, or smoke for 3 hours before testing and to wear comfortable shoes and loose-fitting clothes. Patients will also be informed to avoid unusual physical exercise before testing. A brief history and physical examination will be performed, and patients will be advised about the risks and benefits of the procedure. A standard 12-lead ECG is recorded; a torso ECG will be obtained in the supine position and in the sitting or standing position. Following a 30-minute baseline period, which includes the ECG and blood pressure recorded in both positions, patients will be instructed on how to perform the test (approximately 2 minutes).

The Modified Bruce protocol consists of two 3-minute warm-up stages at 1.7 mph and 0 percent grade and 1.7 mph and 5 percent grade. The speed and percentage grade of the modified Bruce treadmill protocol is changed every 3 minutes in the following manner:

| MPH | 1.7 | 1.7 | 1.7 | 2.5 | 3.4 | 4.2 | 5.0 | 5.5 |
| --- | --- | --- | --- | --- | --- | --- | --- | --- |
| % Grade | 0 | 5 | 10 | 12 | 14 | 16 | 18 | 20 |

Heart rate, blood pressure, and ECG will be recorded at the end of each stage of exercise, immediately before and immediately after stopping exercise and for each minute for at least 5 to 10 minutes in the recovery phase.

### Ambulatory Blood Pressure Monitoring

The ambulatory blood pressure procedure will be similar to that previously published by the PI Dr. Schneider and colleagues (163) and used by Co-PI Dr. Randall and colleagues, and has been pilot tested for the current proposal (cf. Preliminary Studies). Subjects will be fitted with a SpaceLabs 90207 ambulatory monitor (Space Labs, Inc, Redmond, WA) after their clinic assessments at baseline and 3 months. Accuracy of the instrument will be calibrated against a mercury sphygmomanometer. Readings will be taken every 20 minutes during the day and every 30 minutes during the night. While there have been suggestions to reduce the monitoring period and the frequency of sampling, studies indicate that sampling at least every 30 minutes over a 24-hour period gives an accurate and reliable assessment of the 24-hour BP profile (164). Subjects will be instructed to follow their normal daily routine and to complete a diary card for each reading (described below). The next morning subjects will return the monitor to the clinic. The stored data will be retrieved via interface connected to a PC-AT microprocessor. Each reading will be checked for physiological validity by computer program using published criteria (163). White and Morganroth (165) have recently assessed the validity of four commercially available monitors compared to intra-arterial BP during rest and exercise. Based on these data and our own experience with the equipment (cf. Preliminary Studies), we have chosen the SpaceLabs Model 90207 because of its validity, reliability, quietness, and practicality.

*ABPM Diary:* Gellman et al. have studied African American and Caucasian mild hypertensive and normotensive subjects with ABPM and report that posture explained a substantial proportion of within subject variability. Because previous methods for logging daily activities, emotions and other behaviors during ABPM have proved cumbersome, time consuming, and have been poorly standardized and complied with, we will employ an automated behavioral diary, modeled after Van Egeren et al. (166), which was employed in our earlier NIH-funded study on BP patterns with stress reduction [Wenneberg, 1997 #1092] (cf. Preliminary Studies). For this, subjects will mark boxes on a computer card to indicate the time of day, location, posture, activity level and mood associated with each blood pressure reading. The cards will be electronically read with an optical card reader (Chatsworth Model 1000) and the data downloaded to a PC-AT where the data will be merged with the ambulatory BP information and subsequently analyzed. Software for the transfer of diary information from optical card reader to microcomputer to run quality checks, score the data, and quantify critical features has been previously described (166). Ambulatory blood pressure will be measured at baseline and **4**-month posttesting.

### Heart Rate Variability

Heart rate variability (HRV) will be assessed noninvasively with Holter monitoring at baseline and three month posttesting. This protocol has been shown to have excellent reproducibility if done under standardized and appropriate conditions, using a combination of semi-automated analysis and expert over-reading (167). This protocol will be conducted as previously described (167), and implemented in the current NIH supported clinical trials in coronary heart disease directed by Dr. Schneider and colleagues (cf. Preliminary Studies).

*Subject preparation.* Patients will continue their standard medication unchanged. A 24-hour period of ambulatory Holter monitoring will be recorded during which subjects will document their activities in a diary. Subjects will be instructed to carry out their usual daily activities, except for bathing. A structured diary will be included to record activity and symptoms.

*Data collection*. The cardiovascular technician will appropriately prepare the chest wall skin site for electrodes at the modified V2 and V5 positions. These positions allow data on all Holter parameters to be obtained. Subsequently, the cassette Holter recorder is attached, and impedance measurements (10 Hz signal) are used to verify a skin preparation < 5000 ohms and preferably < 35000 ohms.

*Data analysis*. A Marquette Series 8000 Laser Holter system will be used to analyze the Holter tapes. Tapes will be read at 500 times real-time and the electrocardiographic data will be digitized at 128 Hz. All QRS complexes during 24 hours will be automatically detected and labeled as one of the following: normal sinus beat, supraventricular or ventricular ectopic beat, artifact, or unclassified. Unclassified responses and artifacts will be manually edited. The Holter recorder permits recording of cardiac arrhythmias, ST-segment changes, the signal averaged ECG (SAECG), and heart rate variability. RR interval functions will be generated using only normal-to-normal RR intervals, with defects of data caused by ectopic beats and artifacts interpolated by cubic spline function.

*Spectral analysis of HRV*. The Marquette instrument permits analysis of the SAECG from localized time periods, and provides time domain and spectral domain heart rate variability (HRV). The program permits detailed analysis of HRV in the time domain on a 5-min, hourly, or 24-hour basis, and spectral domain data (FFT) on hourly or 24-hour basis. In addition, a 24-hour spatial (Power, Hz, time) portrayal of the spectral data is also provided. RR intervals during, or 15 minutes after, any transient myocardial ischemic episode, which is defined as ST-segment deviation from baseline >1 mm for > 1 minute, will be omitted.

*Time domain analysis*. RR interval data will be transferred to a PC for additional analysis of HRV in the time domain using the Porges method (168) to extract heart period, vagal tone, and low frequency tone. The Porges method has been found to be less susceptible to the erroneous contribution of non-periodic contributions to variance in the low frequency band, and will be used as a methodological control for the estimate of both RSA and low frequency tone. The Porges vagal index quantifies the amplitude of respiratory sinus arrhythmia using the following processes: (1) heart period is time-sampled every 500 ms; (2) a 21-point moving cubic polynomial filter is stepped through the time sampled series to produce a smoothed template series; (3) a digital bandpass (25 coefficients) extracts variance in RSA band (.12-.40); and (4) the natural log of the transformed series results in the index of vagal tone. A similar procedure is done for low frequency tone. However, a 51-point polynomial is used and the bandpass filter is set at .06-.10 Hz. The amplitude of high-frequency component data will be averaged over every minute, and the 1-minute averaged amplitude of high-frequency component data will be averaged over time awake and time asleep based on diary notation. Values averaged over 24 hours, time awake, and time asleep will be considered separately.

### Behavioral Risk Factors

The following CVD behavioral risk factors and psychosocial stress factors will be assessed at baseline and **4**-month posttesting, prior to laboratory testing.

#### Exercise

Physical activity will be measured using a digital pedometer (Omron model HJ-112) that will collect data on steps and miles walked, time and distance and total caloric consumption. Each subject will be given a pedometer to wear over a 24 hour period at baseline and 4 months posttest corresponding with the two clinic visits for 24 hour testing of ambulatory BP. In addition to the pedometer data as a measure of daily physical activity, a modified Minnesota Physical Activity Questionnaire (169) will also be used to assess frequency, duration, type and intensity of home exercise recall over longer time periods. This information will be converted into “physical activity points,” with each point comparable to 4 kilocalories of energy expenditure.

#### Diet

Dietary habits of each group will be assessed and compared. The Block Dietary food consumption questionnaire (130 items) will be used to measure food and nutritional supplement intake for all subjects at pretest and posttest. Developed by Dr. Gladys Block of the National Cancer Institute (170), this full-length questionnaire has been continually updated and tested for validity and reliability (171, 172). It has been used by over 700 research and public health groups including the NHANES survey. Both type and quantity of the major food groups are included. The questionnaires will be reviewed for subject completeness by the clinic staff. Dr. Gladys Block and associates at the Berkeley Nutrition Services, Berkeley, CA will then analyze them for major nutrients, including fats, vitamins, antioxidants, and minerals.

#### *Substance Abuse:* Smoking and Alcohol Use

The Timeline Followback (TLFB) is an estimation method developed to aid in the recall of daily drinking which has been used widely with both African Americans and whites (173) and has been shown to be sensitive to behavioral interventions in the Project MATCH trial. TLFB yields more precise drinking estimates than simple quantity/frequency method. It has high test-retest reliability and criterion/contact validity; that is, it significantly correlates with concurrent variable events, measures of alcohol-related consequences, and biochemical measures (173). The TLFB method is now also used with smokers in clinical settings and appears to display similar psychometric properties under these conditions (173, 174).

### Body Mass Index and waist-hip ratio

Body weight will be measured with a beam-balance scale without shoes or outdoor clothing. Height will be measured with the scale's rule. Since Adams-Campbell and colleagues (175) reported that body mass index (BMI) as a general indicator of obesity is a significant predictor of BP in African Americans (better than waist-to-hip ratio), BMI (kg/m^2^) will be calculated for each participant. Waist to hip ratio will be calculated as waist circumference (at narrowest point between ribs and iliac crest) divided by hip circumference (at the maximal buttocks).

### A.4.8. Metabolic system markers

After an overnight fast, plasma samples for total cholesterol, HDL, glucose, insulin and glycosylated hemoglobin will be assayed at baseline and 4 months posttest by standard reference methods by the Howard University General Clinical Research Center laboratory (GCRC). Insulin will be assayed by radioimmunoassay and glycosylated hemoglobin by affinity chromatography.

#### A.4.9 Cortisol and DHEAS

Alterations of the hypothalamic-pituitary-adrenal (HPA) axis are associated with psychosocial stress, depression, and cardiovascular disease (85, 86). Urinary cortisol (12 hour collection periods) will be measured by high pressure liquid chromatography (HPLC) and plasma/serum DHEA-S will be measured by ELISA through the Howard GCRC. Both cortisol and DHEAS will be measured at baseline and four month posttest.

**A.4.10 Cardiovascular markers**

Aldosterone will be assayed at baseline and 4 months posttest by the Howard GRC.

| A.4.11 Somatotrophic (Growth Hormone/IGF-1) Axis  Plasma samples for IGF-1 (insulin-like growth factor-1) will be assayed at baseline and 4 months posttest by the Howard GCRC using the radioimmunoassay. |
| --- |

**A.4.12 Immune system marker**s

Neuroimmune dysregulation and inflammation may mediate relationships between psychosocial stress, e.g. depression and HF (85). Therefore, high sensitivity C-reactive protein (hs CRP) will be measured as an index of inflammatory activity at baseline and four month posttest. Venous serum samples will be analyzed by high-sensitivity ELISA assay. Fibrinogen will be assayed from plasma using the Clauss method. Measures for both markers (CRP and fibrinogen) will be taken at baseline and 4 months posttest through the Howard General Clinical Research Center.

### A. 4.13 Psychosocial Factors

While a number of psychosocial factors such as anger, hostility, depression, and anxiety have been associated with hypertension, we have chosen to measure anger expression—anger-in, anger-out, and anger-control—in this mechanistic study because of its clear association with hypertension in African Americans (15, 18, 19, 21, 31, 176-180).

a. *Anger Expression (AX) Scale* (181) (24-item version) with three subscales to evaluate Anger-In, Anger-Out, and Anger-Control (alpha = .73-.84). African Americans who suppress anger when provoked, or express anger without reflection, have higher resting BP than those who express anger only after reflection (35). Anger suppression has been shown to correlate more strongly with high BP in African American students than other risk factors (182) and anger expression was associated with more reported chronic health problems, independent of traditional risk factors, in a National Survey of Black Americans data set (20). Both high levels of anger-in and anger-out as measured by the Spielberger AX scale predicted hypertension development at 4 years (180). Each 1-point increase in Anger-out was associated with a 12% increase in risk for hypertension after 4 years of follow-up. This same relationship was also found for Anger-in (180). Anger inhibition has also been found to related to higher ABP, independent of perceived racism (179). Anger expression has been found to be an important factor in the recovery process from a laboratory stressor (183). The combination of outward anger expression and hostility also has been associated with total peripheral resistance and cardiac output (184).

### A.4.14 Demographic Variables

In addition to the primary and secondary outcomes of the study, we will gather data on demographic variables such as age, gender, SES (education and income levels), occupation, marital status, and family history of cardiovascular disease at baseline. Variables will be entered as possible control variables in our analyses if differences between groups are discovered at baseline.

### A.4.14 Medication Usage

All prescription and nonprescription medications will be recorded for dose, frequency and duration by a standardized method. Subjects will be asked to bring their medicine bottles to each laboratory testing session for the study staff to confirm these data at baseline assessment and again at **4**-month posttesting. Information on drug name, and prescribed dosage will be recorded by the research assistant and subjects will be surveyed regarding their compliance with their medication regimens. Using this information, levels of medication intake will be analyzed for the following drug classes: ACE inhibitors, angiotensin II receptor antagonists, beta blockers, calcium channel blockers, central adrenergic agents, diuretics and vasodilators. As the basis for this analysis, an index of medication intake by class of drug will be created in the study database as follows. First, the total daily prescribed dose for each drug will be expressed as a ratio of the minimum recommended dosage level for the drug as reported in the Physicians Desk Reference. Second, these ratios will be summed across all medications in each class of drug, to indicate level of medication usage within each drug class. Medication levels will be included in the statistical analysis as described in D.6.2.

### A.4.15 Compliance

We will assess treatment compliance throughout the intervention period for both the experimental and control groups. In addition to having the same number of group treatment sessions (cf. Intervention section below), subjects in both groups will practice their respective Transcendental Meditation and health education programs at home for approximately 20 minutes twice a day. For the health education group, subjects will be given a list of suggested activities that they will be encouraged to practice at home, including listening to music, reading a book, healthy cooking, and walking or other physical exercise.

We will use multiple methods to triangulate data and arrive at an assessment of treatment compliance. (235-237) These will include:

- 1. percentage of group instructional and follow-up treatment meetings attended,
  2. subject responses to a questionnaire administered at **4**-month posttesting, indicating the percentage of time the assigned program was practiced at home over the most recent two-week period, and
  3. an Ecological Momentary Assessment (EMA) method that will provide a more objective evaluation of the extent to which subjects are practicing their stress reduction or health education program at home. Our EMA procedure will involve having all subjects report on average every two weeks throughout the course of the study on whether they practiced or not their assigned program with respect to a specific morning or evening period on a specific, randomly-designated day. Project staff will call a given subject either in the morning or evening and ask the subject whether he/she practiced the assigned program for that specific time period. This EMA procedure will serve to reduce distortion caused by recall bias by assessing compliance with treatment through reporting of immediate experience (238).

For each of these 3 measures, a 50% threshold will used to determine compliance. In addition, a composite index, made of the average of the percentages of these 3 measures, will be used as a more global assessment of compliance.

In order to obtain a further understanding of the convergence and divergence of the data (235), we will do the following. Concurrent validity of all assessments of compliance then will be determined by examining the correlation among the various measures by treatment group. Correlations of each compliance measure across different months will be used to evaluate time stability. In addition, each of the assessments as well as the composite index, described above, will be used as predictors of change in the primary outcome.

### A.4.16 Social Desirability:

Short-form Social Desirability Response Scale (185) (Cronbach alpha = .69) is based on the Crowne-Marlow scale (186) that has been used widely to control statistically for a “social desirability” response set in African American and white populations. Because respondents to self-report scales often answer questions in a way that represents them favorably, it is considered important to control for such social desirability responses.

### A.4.17 Expectancy*:*

Expectation of benefits will be assessed by a brief questionnaire used in our previous clinical trials in similar populations (126, 187). Expectancy of benefits may affect BP outcomes independently of direct effects of the interventions (188).

### A.4.18 Data Management and Quality control Procedures

All data collected at the field site at Howard University Medical Center in Washington, DC will be sent to the Center for Natural Medicine and Prevention—Data Management and Biostatistics Unit at MUMRI, directed by Drs. Nidich and Rainforth. Data will be entered into an MS ACCESS database, which presently stores data from over a dozen NIH-sponsored research studies. A manual of operations developed by the Data Management and Biostatistics Unit to maintain quality control will include specific procedures for administration of measures and data collection at the field site, transfer of data from field site, checking of data for completeness and authenticity, clerical form handling and filing, data storage both on-site and off-site, data tracking procedures, data cleaning, importation of data into statistical software programs, the monitoring of adverse events, and most importantly procedures for maintaining participant privacy and confidentiality at all levels of the data collection, entry, management, and analysis stages. These procedures, which are being used in other current NIH-sponsored trials, will be applied to the proposed study (cf. Preliminary Studies).

The error detection process will include computerized range and code checks and visual checking procedures (189, 190).

## Interventions

### Common Design Issues for Experimental and Control Interventions

The intervention program was designed in consideration of rigorous clinical research requirements (191, 192) and modeled after previously piloted and standardized intervention methods of NIH-sponsored controlled trials of CVD prevention with nonpharmacological approaches in diverse ethnic and age populations (193), the Treatment of Mild Hypertension Study (TOMHS) (194), as well as our own experience in implementing NIH-sponsored CVD prevention trials with behavioral interventions in high-risk African American populations over the past 15 years (cf. Preliminary Studies). The first objective is to match the experimental and control groups fo**r time and attention. Fo**r this purpose, the health education control intervention is designed to control for contact hours with the instructor, class and home participation, expectancy of positive benefit for disease prevention and health promotion, group size, cultural sensitivity, and instructor qualifications, gender, and ethnicity. The two interventions will follow the same general format of instruction as was used successfully in TOHP I and II with minority samples (193) and standardized and validated in our previous studies (cf. Preliminary Studies). All subjects will be monitored by Co-PI Dr. Randall for their BP status and will continue with their usual medical care provided by their primary physician..

### Structure and Contact Pattern

The two intervention arms will have similar group contact schedules including weekly follow-up meetings. Both groups will receive similar health education instruction over the course of the 4 month study period. In addition, the experimental group will receive instruction in the Transcendental Meditation program in separate sessions (i.e. at different times and places) from the health education sessions. The health education alone control group will receive additional hours of group contact in the form of educational films and other educational activities to match the experimental intervention for time and social support. Overall, both groups will receive equal hours of attention and time from their intervention instructors.

Follow-up meetings of the intervention (i.e., duration of treatment phase) will be held both for the experimental and control groups to maintain participation in the treatment. Attempts will be made to re-engage inactive participants. These sessions will promote contact and adherence to the interventions (196).

All participants will be informed of their group assignment, including the time and place of their first intervention meeting, by a research assistant who will not be involved in data collection or intervention implementation. Participants may need to wait one to three weeks between enrollment and their first group meeting while the project accrues sufficient numbers of subjects for groups. During this time the project manager will maintain regular contact with participants to maintain enthusiasm and commitment to the study.

### The Transcendental Meditation Program

The Transcendental Meditation program was chosen from among a variety of available stress-reduction meditation practices because of its systematic and repeatable teaching protocol, feasibility, reproducible physiological results, and distinctive clinical effectiveness as reported in previous comparative studies, clinical controlled trials, and meta-analyses (195, 197-199). (Please see manual located in appendix for a more complete description of the Transcendental Meditation program intervention.) The Transcendental Meditation technique is a traditional meditation modality that has its origin in the ancient Vedic tradition (200). Maharishi Mahesh Yogi is credited with reviving and restoring this meditation practice in accordance with classical Vedic texts (201). The Transcendental Meditation technique has been taught worldwide since 1957. Approximately two million people in the US and six million people worldwide have participated in the standard Transcendental Meditation course over the last 45 years (195). Qualified and experienced African American Transcendental Meditation teachers in the Washington, DC area have been implementing this intervention in clinical trial in the Washington, DC area. These teachers will be available for the current study.

*Systematic and reproducible*. Recent guidelines from the National Institutes of Health and other authorities recommend that traditional health promotion practices, including meditation, be studied in their intact form without dismantling and potentially disrupting their integrated and interdependent components (202, 203). Therefore, the current behavioral medicine protocol will employ Transcendental Meditation as systematically taught throughout the world in its original form. In contrast to other mind-body techniques, both traditional and modern, the Transcendental Meditation technique is highly reproducible, as described below (195, 204). Standard pre-printed teaching materials will be used throughout the course to ensure quality control and replicability.

*Distinctive effectiveness*. It is often maintained that a wide range of meditation and relaxation techniques produce similar results, but recent comparative studies and systematic reviews do not confirm this generalization (198). Specifically, a series of published meta-analyses comparing different relaxation and meditation techniques across approximately 300 independent experimental samples at numerous study sites report that Transcendental Meditation practice, compared to other approaches to stress reduction, is consistently associated with significantly greater reductions in physiological arousal (124) trait anxiety (205), and smoking and drug abuse (199) and with significantly greater enhancement of psychological health and maturity (206). In addition, the present investigators' preliminary studies with high-risk African American subjects have demonstrated relatively high ease of implementation, personal and cultural acceptability, and compliance rates (126, 127).

#### Outline of Transcendental Meditation Instruction and Follow-Up Program

The core instruction in the Transcendental Meditation technique will involve a seven-step course over six days, which will follow the standard format offered throughout the U.S. by Maharishi Vedic Universities and Maharishi Vedic Schools (195): a) Introductory Lecture—a review of previous scientific research on the Transcendental Meditation program and a vision of possible benefits through practice; b) Preparatory Meeting—a review of the mechanics and origin of the Transcendental Meditation technique; c) Personal Interview—interview with a qualified teacher of the Transcendental Meditation program; d) Personal Instruction—individual learning of the Transcendental Meditation technique; e) First Day of Checking—verifying the correctness of the practice and further instruction; f) Second Day of Checking—understanding of the mechanics of the Transcendental Meditation technique from personal experiences; g) Third Day of Checking—understanding the mechanics of the development of higher states of wellness and health. Most sessions will last 1 to 1.5 hours with the exception of the personal interview (about 10 minutes). The general format of most sessions will be lecture/discussion

Follow-up Program: Following this initial phase of the intervention, there will be follow-up program twice a month for the duration of the four-month intervention period that will include: a) checking of correct practice of the Transcendental Meditation technique and b) advanced lectures and seminars to ensure complete understanding of benefits of the practice for physiological, psychological, and behavioral health. Follow-up sessions will be structured according to the staged contact schedule described above. Please note that the standardized Transcendental Meditation program intervention will not include analysis of contemporaneous or historical stressful experiences. Instead the instructional program will focus on correct practice of the Transcendental Meditation technique regularly for deep rest, relaxation, and enhancement of neurophysiological homeostasis. Specific stressors will be discussed only in terms of their potential impact on regularity of practice (195, 200).

A.5.4. Additional Educational Program for Control Group to Match for Core and Follow-up Sessions of the Transcendental Meditation Program.

The control group, in addition to the standard health education program (see section A.5.5) described above, will have a series of sessions that match the time of core instruction and follow-up meetings the Transcendental Meditation program. These additional sessions will include educational films, guest speakers, and other instructional media that engage the participants, without specifically providing new health education content.

### A.5.5 Health Education (HE) Program

Both groups will receive a course of didactic health education twice a month on the prevention of CHD through lifestyle modification of conventional CHD risk factors (e.g., high blood pressure, serum cholesterol, smoking, weight, physical inactivity) (207, 208). This program will be similar in structure and content to the health education control programs successfully implemented in our previous NIH-sponsored trials on prevention of CVD in high-risk African Americans (126) (cf. Preliminary studies) and is modeled on the published protocols and operations manual of the Trials of Hypertension Prevention (TOHP I, TOHP II) (209, 210). Subjects will learn about the value and approaches to making and maintaining reductions in primarily body weight, dietary fat and salt. The saturated fat, cholesterol, and sodium reduction segments will be event-oriented, emphasizing knowledge and approaches to controlling dietary intake in a variety of situations. Participants will learn to identify sources of fat and sodium in their food and the value of planning, reading labels, shopping and modifying recipes. The weight management section will focus on the value, knowledge and approaches to decreasing caloric intake, counting fat intake, and increasing physical activity. Participants will learn to identify major sources of high calorie foods and make appropriate substitutions. Participants will learn the value of mild to moderate intensity physical activity on a daily basis. The sessions will include discussions of stress as it relates to weight management and physical exercise. By design, however, and to avoid confounding the experimental design, the control health education sessions will not include instructions in any specific stress reduction or relaxation techniques.

### A.5.6 Quality Assurance Measures

A detailed Manual of Operations for both interventions will be developed and strictly adhered to. In order to assure a high quality of the interventions and a standardized approach to delivering the intervention, the Manual of Operations will address the following issues regarding treatment quality assurance: 1) selection of individuals who provide the intervention, 2) certification of instructors, 3) use of standardized instructional materials and standardized instructional sessions, 4) cultural sensitivity, 5) observation of instructors, 6) record keeping of small group sessions, 7) small group size, and 8) on-going assessment of participant treatment compliance for at-home practice and class attendance. A copy of the Protocol Manual for the meditation intervention is included in the Appendix. Also refer to Preliminary Studies for pilot testing of the experimental and control interventions and study procedures.

## DATA ANALYSIS

### Sample Size Determination

The sample size is calculated by statistical power analysis (211) to provide sufficient power to detect clinically meaningful change in clinic BP as the primary outcome. Based upon average BP changes reported in meta-analyses of other behavioral treatments (including dietary modification, exercise, weight loss, and alcohol reduction) (212-215), we determined that a decrease of -4.5 /-3.0 mm Hg SBP/DBP would be clinically meaningful, and based upon our prior research showing a decrease of -10.7/-6.4 mm Hg SBP/DBP (126), we anticipate a treatment effect at least this large. For the sample size calculation, standard deviations at baseline were obtained from our 3-month trial of meditation in the treatment of hypertension in African Americans (126, 127)(cf. Preliminary Studies): 13.4 mm Hg for SBP and 8.7 for DBP. These values are quite similar to standard deviations averaged over several other trials in mild hypertensives (212, 213, 216). Again using the Oakland trial (126, 127), we assumed a test-retest correlation in BP scores of .65 over 3 months. Based upon repeated measures ANCOVA of monthly BP levels using two-sided tests at the 5% significance level and 90% statistical power, and allowing for 15% attrition, we determined that 76 subjects per group would be needed for SBP and 72 subjects per group would be needed for DBP. Hence 76 subjects per group would provide sufficient statistical power for both clinic BP outcomes. Thus a total of 152 subjects need to be recruited to reliably detect a difference between Transcendental Meditation and health education controls on the primary outcome.

Published data indicates that this sample size of 152 will also provide adequate power to detect clinically meaningful changes in ambulatory BP and blood pressure reactivity outcomes. With regard to ambulatory BP, we used baseline SD’s of 13/10 mm Hg for SBP/DBP and test-retest correlations of .79 and .66 that were reported in two studies in African Americans (179, 217). Assuming ANCOVA on change from baseline to 4 months with an alpha level of .05, and allowing for 15% attrition, we calculate that our sample size will provide 80% power to detect a decrease of –4.0/-3.8 mm Hg SBP/DBP in ambulatory BP as statistically significant. For the blood pressure reactivity calculation, we used pre-treatment means and SD’s for delta scores obtained during mental arithmetic and cold pressor tasks and exercise stress (76, 218, 219) and test-retest correlations from a meta-analysis on reproducibility of BP reactivity (220). Using this data and assuming ANCOVA on change from baseline to 4 months with an alpha level of .01 (see D.6.2 below), we calculate that a sample size of 152 subjects would provide at least 80% power to detect attenuations due to treatment in BP responses of –5.1/-4.6 mm Hg during mental arithmetic, -6.9/-5.1 mm Hg during cold pressor, and –8.4/6.9 mm Hg during exercise stress. We note that previous research found attenuations in BP reactivity of similar magnitudes in African Americans and whites with the same experimental treatment as our proposed study (32, 221).

To determine the statistical power to detect a mediator effects, we assume it desirable to have adequate power to detect a medium sized mediator effect. Following MacKinnon’s criteria for a medium-sized mediator effect and methodology for comparing tests for mediators (229), we posited α=β=.39, and performed a Monte Carlo simulation of 1000 randomly generated datasets of 152 subjects. This analysis indicated that Mackinnnon’s asymmetric confidence limit test for mediators would provide 91% power to detect a mediator using the .01 significance level in our proposed study.

### Statistical Analysis

All patients will be analyzed according to the treatment group into which they were randomized, i.e., by intention-to-treat analysis (222), using the SAS (version 9) statistical package. We will use a standard method of multiple imputation for missing values. In this approach, multiple data sets are created with a missing data point imputed with a value chosen at random from the distribution of the variable of interest. After n complete data sets are generated, they are analyzed separately using classical statistical methods for complete data, and then the distribution of results for the imputed datasets are summarized to estimate a p-value for the original dataset. (This process is handled automatically in SAS PROC MI/ MIANALYZE.) Hence, statistical analysis will be performed on all subjects who are randomized (N=152), including dropouts.

Covariates used in all analyses of treatment outcomes will be prespecified as follows. Analysis of change in each outcome will be based upon covarying for the relevant pretreatment baseline score. Additional covariates will be pretreatment values for age, gender, baseline blood pressure, and diuretic medications. Other baseline variables will not be used as covariates because randomization eliminates bias in the assignment of subjects to treatment groups; randomization implies that any apparent between-group differences on baseline variables that might occur will merely reflect sampling error (230). In addition to the main analyses of the study outcomes, the potential confounding influence of treatment compliance and changes in antihypertensive medication levels will be assessed by including these variables as additional covariates in ancillary analyses of the outcomes. If treatment remains significant as the independent variable in these ancillary analyses then this will indicate that the treatment effect is not due to these potential confounds.

For analyses of change in clinic and ambulatory BP outcomes, an alpha level of .05 will be used. For all secondary outcome analyses, an alpha level of .01 will be used in order to provide a conservative test of treatment effects. A modified Bonferroni adjustment was considered but ruled out because it would be overly conservative in this context (223).

The statistical analysis plan to achieve the specific aims of the study is as follows:

**1. To confirm effects of meditation compared to control on clinic blood pressure**

Changes in clinic SBP and DBP levels will be analyzed separately by repeated measures analysis of covariance (ANCOVA), with BP **a**t 1, 2, 3 and 4 months follow-up as the dependent variables, and treatment as the grouping variable. Mean change in BP levels will be adjusted by covarying forbaseline BP (averaged over the last two baseline visits) and other prespecified covariates as described above.

**2. To demonstrate effects of meditation compared to control on ambulatory blood pressure**

Data on levels of ambulatory blood pressure (AMBP) will be adjusted for the potentially confounding effects of posture, activity level, location and mood retrieved from the computerized diary record. A mixed effects repeated measures model will be used to analyze the effects of treatment and daily variations on ambulatory BP (224, 225). In this statistical model, treatment group and testing time (pretreatment vs. post-test) will be entered as fixed effects, while subject ID and daily variations will be considered as random effects. Age, gender, baseline blood pressure, and diuretic medications will be used as covariates. Testing time will be the repeat measures factor and treatment will be the grouping variable. To assess the effect of treatment on nocturnal BP decline (“dipping”), BP levels averaged over daytime and nighttime hours will also be analyzed using a mixed model repeated measures analysis. In this analysis time of day (daytime vs. nighttime) and subject ID will be considered as random effects and the fixed effects will again be treatment group and testing time.

**3. To evaluate effects of meditation compared to control on cardiovascular reactivity to and recovery from a battery of psychological and physical stressors**

Cardiovascular responses in BP and HR to the three stressor tasks—mental arithmetic, exercise treadmill, and cold pressor—will be analyzed in the same way.

Effects on BP and HR reactivity: For each variable, reactivity will be analyzed in terms of “delta scores”—defined as change from the baseline period preceding the stressor task to the mean level during the stressor. Treatment effects on reactivity will be analyzed by repeated measures ANCOVA, in which the grouping variable will be treatment (Transcendental Meditation vs. HE), the repeated measures factor will be time segments during the stressor period, the dependent variables will be the delta scores for the outcome measured during the stressor at **4-**month post-test, and the corresponding delta scores measured at pretreatment will be the time-dependent covariate. The main effect of treatment in the repeated measures ANCOVA model will indicate a difference between the groups on change from pretreatment to post-test with regard to the mean level of reactivity during stress (87).

Measurement of BP and HR recovery: Recovery from laboratory stressors will be measured after each stressor period. The approach we will use is based on fitting a 3-parameter mathematical curve for each patient to the series of readings taken during recovery. Parameter “a” represents the amount that the measure drops between the stressor level and the post-stress recovery level; parameter “b” represents the time it takes the measure to drop from stressor level to the recovery level; and parameter “c” represents the post-task recovery level, relative to baseline (99). The estimated values of the a, b, and c parameters will be used as dependent variables in multivariate ANCOVA models to assess the differences among the conditions related to treatment. Results for the three parameters together will indicate the broad latent construct of "blood pressure recovery".

**4. To evaluate effects of meditation compared to control on cardiovascular hemodynamic functioning**

Changes in cardiac output, stroke volume, total peripheral resistance, and arterial compliance will be separately assessed by ANCOVA’s, with change from baseline to posttest at **4** months as the dependent variable, and treatment as the grouping variable. Prespecified covariates, including the pretreatment level will be prespecified as described above.

**5. To evaluate effects of meditation compared to control on autonomic nervous system tone**

Changes in measures of autonomic tone--low frequency power, high frequency power, and standard deviation of heart period—will be assessed by separate ANCOVA’s, following the same method as for Aim 4. Changes from pretreatment to posttest in resting levels of plasma catecholamines and changes in catecholamine response to each laboratory stress (defined as the catecholamine level during stress minus the level during the resting baseline) will be analyzed by the same ANCOVA method..

**6. To evaluate effects of meditation compared to control on behavioral and psychosocial risk factors**

To assess differences in treatment conditions, statistical analyses will be performed on the following: 1) lifestyle CVD risk factors—cigarette consumption, alcohol consumption, physical activity, and body mass index; 2) dietary components—total calories, fats, sodium and potassium; and 3) anger expression (anger-in, anger-out, anger-control, total score). Each outcome will be assessed by ANCOVA, with posttest at 3 months as the dependent variableand treatment as the grouping variable. Prespecified covariates, including the pretreatment level will be prespecified as described above.

**7. To investigate possible mediators by which meditation may modify clinic and ambulatory blood pressure.**

We will test the significance of intervening variables as potential mediators of the treatment on blood pressure changes using the asymmetric confidence interval test developed by MacKinnon (229,231) Monte Carlo simulation studies (229,231) have shown that the asymmetric confidence interval test properly controls for type I error and has superior statistical power than 13 other published tests for mediator effects, especially compared to causal steps methods (e.g., Baron & Kenny (1986) (232, 239) and also compared to LISREL-type structural equation model analysis (233). MacKinnon’s approach is a refinement of the standard Baron and Kenny (1986) approach to testing for mediators in that it utilizes information from regression coefficients from statistical models concerning the relationship between the treatment variable (T), the mediator variable (M), and the outcome (Y). Specifically, asymmetric confidence interval test is based on the product of regression coefficients αβ (where α is the regression coefficient of T on M, and β is the regression coefficient of M on Y while covarying for T), which represents the strength of the mediator effect. MacKinnon’s method uses the bias-corrected bootstrap method (234) to construct confidence intervals in order to reject or retain the null hypothesis that the mediator effect αβ =0. Statistical power is enhanced compared to other methods because statistical inference is accomplished in a single step, and the nonnormal sampling distribution of estimators of αβ is explicitly taken into account. We will conservatively adopt the .01 significance. The outcome variables in Aims 3-6 will be tested as potential mediators of treatment effects separately for clinic BP and ambulatory BP, Aims 1-2.
