# Supplementary material for: Effects of Lifestyle Modification on Telomerase Gene Expression in Hypertensive Patients: A Pilot Trial of Stress Reduction and Health Education Programs in African Americans
Source: PLoS One. 2015 Nov 16;10(11):e0142689. doi: 10.1371/journal.pone.0142689 (PMC4646647; doi:10.1371/journal.pone.0142689)
Supplement: S1 Table — (DOCX) [file pone.0142689.s003.docx]

**S1 Table:** Inclusion of subjects who participated in the telomerase substudy compared to those who did not

| **dependent variable** | **N**  **group 0** | **mean**  **group 0** | **SD**  **group 0** | **N**  **group 1** | **mean**  **group 1** | **SD**  **group 1** | **p value** |
| --- | --- | --- | --- | --- | --- | --- | --- |
| male | 104 | 27.9% | 45.1% | 48 | 47.9% | 50.5% | 0.02 |
| age | 104 | 58.9 | 10.2 | 48 | 58.2 | 10.7 | 0.68 |
| systolic BP | 104 | 146.3 | 6.3 | 48 | 145.9 | 5.2 | 0.71 |
| diastolic BP | 104 | 84.7 | 7.5 | 48 | 85.2 | 7.7 | 0.70 |
| telomere length | 104 | 84.7 | 7.5 | 48 | 85.2 | 7.7 | 0.70 |
| weight | 103 | 88.5 | 18.2 | 48 | 86.1 | 15.6 | 0.43 |
| BMI | 103 | 32.4 | 6.4 | 48 | 30.0 | 5.3 | 0.03 |
| anger in score | 103 | 6.0 | 3.9 | 48 | 5.7 | 4.1 | 0.60 |
| anger out score | 103 | 6.6 | 2.9 | 48 | 6.5 | 2.8 | 0.86 |
| anger control score | 103 | 13.6 | 3.6 | 48 | 13.3 | 3.7 | 0.70 |
| anger total score | 103 | 23.0 | 7.9 | 48 | 22.8 | 7.5 | 0.88 |
| moderate activity per wk | 88 | 166.2 | 154.5 | 42 | 136.3 | 135.9 | 0.29 |
| vigorous activity per wk | 88 | 62.8 | 101.4 | 42 | 57.4 | 86.2 | 0.77 |
| sodium | 82 | 1996.9 | 1270.5 | 38 | 2205.8 | 1240.6 | 0.40 |
| carbohydrates | 82 | 188.1 | 89.6 | 38 | 216.3 | 121.9 | 0.16 |
| protein | 82 | 60.8 | 48.2 | 38 | 62.5 | 36.2 | 0.85 |
| fiber | 82 | 17.8 | 8.6 | 38 | 17.5 | 8.7 | 0.86 |
| calories | 82 | 1614.8 | 914.1 | 38 | 1807.0 | 1008.2 | 0.30 |
| oleic acid | 82 | 27.0 | 17.4 | 38 | 29.2 | 18.4 | 0.52 |
| linoleic acid | 82 | 15.6 | 11.5 | 38 | 16.1 | 9.4 | 0.82 |
| total fat | 82 | 69.5 | 45.9 | 38 | 76.0 | 47.5 | 0.47 |

| Group 0: Not included in Telomerase substudy | |
| --- | --- |
| Group 1 :Included in Telomerase substudy |  |
